# Integration of genetic and epidemiological data to infer H5N8 HPAI virus transmission dynamics during the 2016-2017 epidemic in Italy

**DOI:** 10.1038/s41598-018-36892-1

**Published:** 2018-12-21

**Authors:** P. Mulatti, A. Fusaro, F. Scolamacchia, B. Zecchin, A. Azzolini, G. Zamperin, C. Terregino, G. Cunial, I. Monne, S. Marangon

**Affiliations:** Istituto Zooprofilattico Sperimentale delle Venezie, Legnaro, (Padua) Italy

## Abstract

Between October 2016 and December 2017, several European Countries had been involved in a massive Highly Pathogenic Avian Influenza (HPAI) epidemic sustained by H5N8 subtype virus. Starting on December 2016, also Italy was affected by H5N8 HPAI virus, with cases occurring in two epidemic waves: the first between December 2016 and May 2017, and the second in July-December 2017. Eighty-three outbreaks were recorded in poultry, 67 of which (80.72%) occurring in the second wave. A total of 14 cases were reported in wild birds. Epidemiological information and genetic analyses were conjointly used to get insight on the spread dynamics. Analyses indicated multiple introductions from wild birds to the poultry sector in the first epidemic wave, and noteworthy lateral spread from October 2017 in a limited geographical area with high poultry densities. Turkeys, layers and backyards were the mainly affected types of poultry production. Two genetic sub-groups were detected in the second wave in non-overlapping geographical areas, leading to speculate on the involvement of different wild bird populations. The integration of epidemiological data and genetic analyses allowed to unravel the transmission dynamics of H5N8 virus in Italy, and could be exploited to timely support in implementing tailored control measures.

## Introduction

Between October 2016 and December 2017, Highly Pathogenic Avian Influenza (HPAI) outbreaks sustained by H5N8 subtype viruses were reported in several European Countries. Cases were identified primarily in wild bird population, with frequent and considerable incursions in the domestic sector, both industrial and rural^1–3^. A HPAI H5N8 strain had been previously reported in 2014–2015, affecting domestic poultry and wild birds in Germany, Hungary, Italy, the Netherlands, Sweden, and the United Kingdom^4^. However, although both the strain circulating in 2014–2015 and the one emerged in late 2016 belonged to clade 2.3.4.4, they could be included into two different genetic groups, with different pathogenicity especially towards wild waterfowl^5,6^. Furthermore, the two groups showed a likely different pattern of spread in wild and domestic birds^1–4^. Out of 18 total cases detected in 2014–2015, 12 notified cases (66.7%) occurred in industrial farms; a single case (5.6%) was reported in a zoo; and 5 positive cases (27.7%) were detected in apparently healthy wild birds (*Anas penelope*, *Anas platyrhyncos, Cygnus olor*)^4^. Contrarily, of the 2821 total H5N8 HPAI outbreaks occurred both in domestic poultry and wild birds between 2016 and December 2017, 1583 (56.11%) were detected on wild waterfowl through passive and/or syndromic surveillance, indicating a likely higher pathogenicity of the virus for wild birds^1–3^. In addition to the H5N8 strain, in 2016–2017 also a HPAI virus subtype H5N5 was detected mainly in wild birds in Europe, and classified as a reassortant of the circulating H5N8. The hemagglutinin (HA) gene segments of the two circulating H5 subtypes were genetically related to H5N8 viruses found in wild birds in Qinghai Lake (China) during May 2016 and in Uvs-Nuur Lake (Siberia) in June 2016. These findings suggested the likely westward dissemination of the virus from Siberia to Europe, via the late-summer/autumn migratory movements of wild waterfowl^5,7,8^.

Italy repeatedly experienced the circulation of both HP and Low Pathogenicity (LP) Avian Influenza viruses in the past two decades. This was mainly due to high concentrations of susceptible poultry production types (e.g. fattening turkeys and laying hens) in a limited geographic areas, defined Densely Populated Poultry Areas (DPPAs) along migratory flight paths (i.e. Black Sea – Mediterranean and The East Atlantic flyways), and in proximity to large wetlands^9^. Nevertheless, after the massive epidemics of HPAI and Low Pathogenicity Avian Influenza (LPAI) viruses in late 1990s and early 2000s, only sporadic incursions with limited outbreaks were observed until 2016^10,11^. Between December 2016 and January 2017, four wild birds found dead in north-eastern Italy confirmed positive for the HPAI H5N5 and H5N8 viruses circulating in Europe at the time. Since then, Italy experienced several HPAI H5N8 cases, both in domestic and wild birds, which could be grouped into two distinct epidemic waves. A first epidemic wave occurred between December 2016 and May 2017. The epidemic started at the fringes of the DPPAs in the north-eastern Regions of Veneto, Lombardy, and Emilia Romagna. The virus was initially detected in two fattening turkey farms and a laying hen premise located in proximity to large wetlands widely frequented by migratory waterfowl^12^. On mid-February, the virus was isolated in two fattening turkey farms located in a DPPA at a distance of about 100 km from each other, which were reported to show increased mortality almost contemporarily. By the end of May 2017 16 outbreaks were observed in poultry farms and seven in wild waterfowl. The epidemic wave followed an atypical pattern of occurrence, with scant weekly reported cases and a limited number of wild birds involved, in sharp contrast with what was reported in other European Countries^13,14^.

The second epidemic wave began on the third week of July 2017 and continued until mid-December of the same year. Sixty-seven outbreaks were observed in poultry farms, while only seven cases were reported in wild birds as caused by the HPAI H5N8 subtype virus. Most of the cases occurred within the DPPA located along the Po river valley in northern Italy, although some isolated outbreaks were reported in western and central regions. This wave was characterized by noteworthy lateral spread, leading to four clusters of secondary cases, with up to 23 interconnected outbreaks.

Hereby we present a detailed description of the Italian H5N8 HPAI epidemic, which proved having interesting characteristics that could help in better understanding the AI spread dynamics in absence of migratory movements, as most of the outbreaks in poultry were detected in Summer/Autumn-time 2017. The integration of epidemiological data and genetic analyses allowed to generate and/or corroborate hypotheses on the evolution of the epidemic, and could be exploited in almost a real-time fashion to support decision-makers in enhancing biosecurity measures and implementing tailored control measures.

## Results

### Epidemiological Investigations

**Figure 1:**
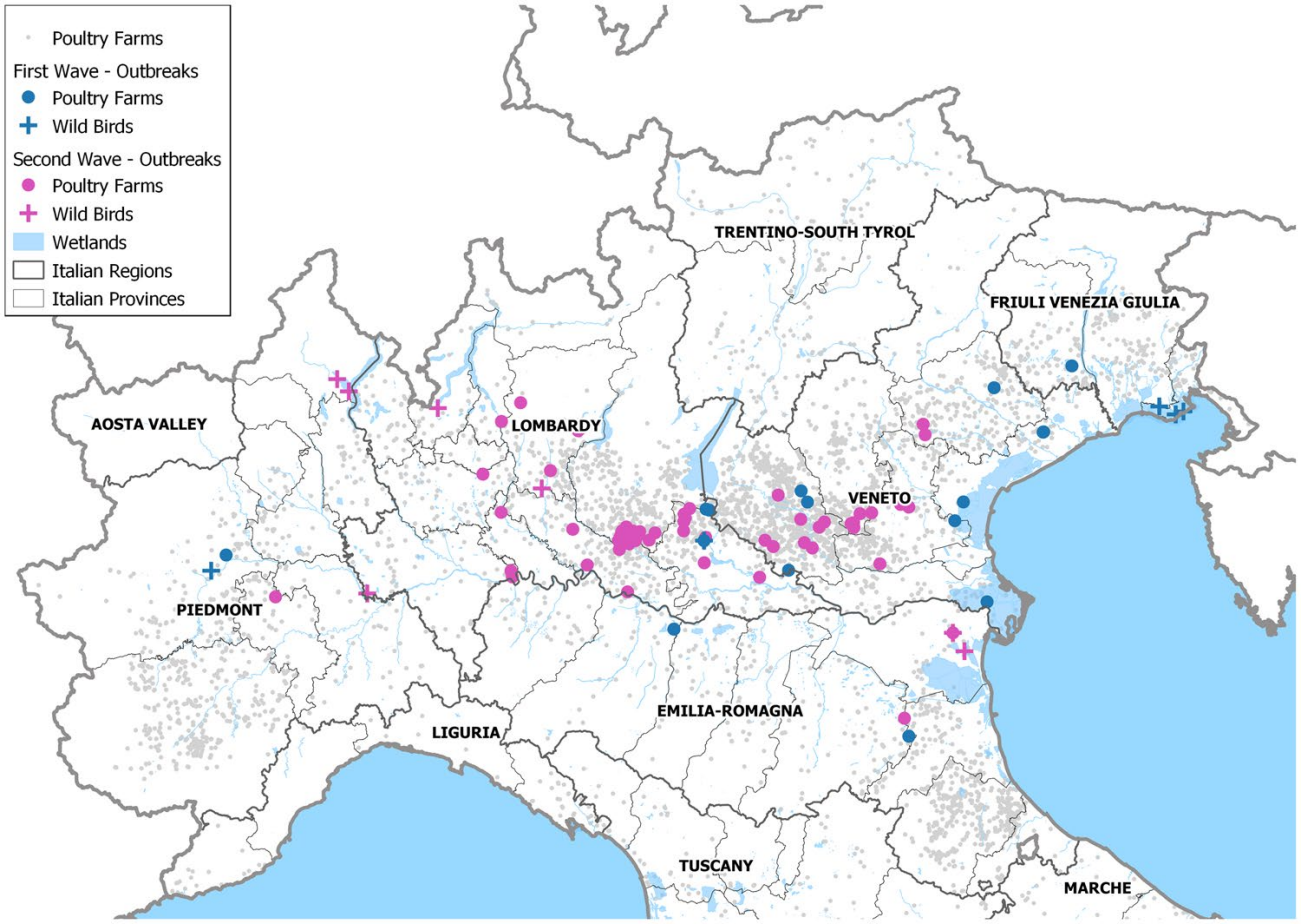
Distribution of H5Nx HPAI cases In Italy in 2016-2017; Cases are classified per epidemic wave (first wave: blue; second wave: magenta), and per type (Domestic poultry: circle; Wild birds: cross).

**Figure 2:**
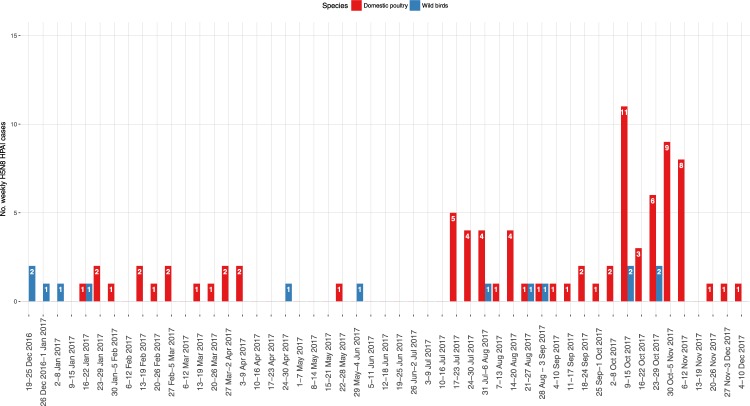
Weekly H5Nx cases in domestic poultry (red columns) and wild birds (blue columns) in Italy in 2016-2017; the dates refer to the onset of symptoms for poultry farms, and finding day for wild birds.

A total of 14 HPAI H5 outbreaks in wild birds (2 H5N5, and 12 H5N8), and 83 poultry farms infected by the HPAI virus of the H5N8 subtype were reported between December 2016 and December 2017, mainly distributed in the DPPAs located in Lombardy and Veneto regions, and in Emilia Romagna (Fig. 1). The epidemic curve described two separate waves, presenting different patterns of outbreak occurrence (Fig. 2). The first epidemic ranged between the end of December 2016 and the end of May 2017, with a total of 16 poultry farms involved, and 7 cases detected in wild birds (Fig. 2 – Table 1). Low numbers of weekly cases were recorded, and several weeks with no cases were observed. The last outbreak of the first wave was confirmed on 30 May 2017, in a fattening turkey farm in proximity of which also a sick juvenile grey heron (*Ardea cinerea*) was found, which was afterwards confirmed being infected with a H5N8 HPAI virus. The most largely affected productive type was fattening turkey (n = 9/16, 56.25%), although from the end of February also backyard farms were involved in the epidemic (n = 5/16, 31.25%) (Table 1).

**Table 1:** Number of H5N8 HPAI cases in domestic poultry in 2017, per Italian Region and productive type.

Wave	Productive types	Affected Regions						*Total cases*
		Emilia Romagna	Friuli Venezia Giulia	Lombardy	Piedmont	Veneto	Lazio	
First	Fattening turkeys	1	0	3	0	5	0	*9*
	Laying hens	1	0	0	0	1	0	*2*
	Rural Farms	0	1	0	1	3	0	*5*
	***Total cases 1st wave***	***2***	***1***	***3***	***1***	***9***	***0***	***16***
Second	Fattening turkeys	2	0	18	0	10	0	*30*
	Laying hens	1	0	7	1	0	0	*9*
	Broilers	0	0	4	0	1	0	*5*
	Geese	0	0	1	0	1	0	*2*
	Game Farms	0	0	1	0	0	0	*1*
	Ducks	0	0	3	0	1	0	*4*
	Rural Farms	0	0	8	0	4	1	*13*
	Chicken Breeders	0	0	1	0	0	0	*1*
	Grower Farms	0	0	1	0	0	0	*1*
	Multi-species	0	0	0	0	1	0	*1*
	***Total cases 2nd wave***	***3***	***0***	***44***	***1***	***18***	***1***	***67***

The first confirmed outbreak of the second epidemic wave was reported on 20 July 2017, with symptoms starting on 17 July 2017. The case was confirmed 50 days after the last infected farm (IF) of the first wave; although both cases were located in the same Italian province, they were located about 30 km apart, no contacts were reported between the farms, and the virus strains isolated in two outbreaks resulted not genetically closely related. During the second wave, 67 IFs were detected in domestic poultry, and the trend of the weekly cases approached a classic epidemic curve, reaching a peak around mid-October, and then decreasing in December (Fig. 2). As in the first epidemic wave, fattening turkey farms were the most affected production type (n = 30/67, 44.78%), although laying hen premises (n = 9/67, 13.43%) and rural farms (n = 13/67, 19.40%) were also considerably involved. By the end of the epidemic wave, five chicken broiler farms (7.46%) were also affected; four of these were located in proximity to each other. Only seven cases in wild birds were reported during the second wave; the most frequently affected species appeared being the mute swan (*Cygnus olor*) (n = 4/7, 57.14%) (Table 2). The last H5N8 HPAI finding in wild birds involved a rock dove (*Columba livia*) and a common kestrel (*Falco tinnunculus*) found on the third week of October, both in proximity to a large layer operation that was confirmed as IF on 5 October, and where the culling, cleansing and disinfection procedures were concluded on 18 October. The viruses found in these two wild birds showed a high similarity (100% for the HA gene and 99.7–99.8% for the complete genome) with the strain identified in the backyard flock of the Lazio Region, although no epidemiological links were detected with other IFs.

**Table 2:** Number of H5Nx HPAI cases in wild birds in 2016-2017, per Italian Region and species.

Wave	Region	Species	Common name	Strain	Confirmation date
First	Friuli Venezia Giulia	*Anas penelope*	Eurasian Wigeon	H5N5	30/12/2016
	Friuli Venezia Giulia	*Anas penelope*	Eurasian Wigeon	H5N8	05/01/2017
	Friuli Venezia Giulia	*Anas strepera*	Gadwall	H5N5	11/01/2017
	Friuli Venezia Giulia	*Cygnus olor*	Mute Swan	H5N8	23/01/2017
	Veneto	*Tadorna tadorna*	Common shelduck	H5N8	23/02/2017
	Piedmont	*Cygnus olor*	Mute Swan	H5N8	29/04/2017
	Lombardy	*Ardea cinerea*	Grey heron	H5N8	07/06/2017
Second	Lombardy	*Anas plathyrhyncos*	Mallard	H5N8	02/08/2017
	Lombardy	*Cygnus olor*	Mute Swan	H5N8	25/08/2017
	Lombardy	*Cygnus olor*	Mute Swan	H5N8	29/09/2017
	Piedmont	*Cygnus olor*	Mute Swan	H5N8	13/10/2017
	Piedmont	*Cygnus olor*	Mute Swan	H5N8	13/10/2017
	Emilia Romagna	*Anser anser*	Wild Goose	H5N8	07/11/2017
	Emilia Romagna	*Falco tinnunculus Columba livia*	Common Kestrel Rock Pigeon	H5N8	07/11/2017

Both the first and the second epidemic waves spread across the most densely populated poultry area in Italy; however, while outbreaks in the first wave were mainly located in the outskirts of the DPPA, most of the IFs in the second wave occurred in the core of the DPPA (Fig. 1). In particular out of 67 cases observed in the second wave, 23 occurred in two adjoining provinces of Lombardy region (n = 22, 95.65%, in Brescia and 1, 4.35% in Cremona) with confirmation dates ranging between 9 October and 22 November, and at an average geodesic distance of 5.97 km (95% CI: 5.67; 6.27 km) from each other. Overall farm density in the affected area was greater in the second wave (F_(1,81)_ = 9.146, p < 0.01), with an average of 50 farms more within a 10-km radius from IFs in the July-December period.

Data on proximity to wetlands and presence of wild waterfowl were recorded in the Epidemiological Investigation form, as indicating potential direct and indirect contacts with wild birds (Table 3). Data were not available for two IFs in the first epidemic wave, and for three IFs in the second wave. Wild birds were observed in proximity to 60.29% of the IFs in the first wave, and in 40.63% of IFs of the second epidemic wave, while the great majority of the IFs were indicated as near wetlands in the whole epidemic. However, when looking at the geodesic distances derived from GIS data manipulation, it resulted that on average IFs in the second epidemic wave were located at a greater distance to wetlands than in the first wave (F_(1,81)_ = 5.485, p = 0.022; Average diff 2^nd^ wave – 1^st^ wave = 2402.73 m, 95% CI: 361.48–4443.99).

**Table 3:** Information on potential direct and/or indirect contacts with wild waterfowl for the first (n = 14) and second (n = 64) epidemic waves; data on the exact distance (m) between farms and wetlands are reported.

Wave	No. IFs with reported wild birds presence [%]^a^	No. IFs reported as in proximity to wetlands [%]^a^	Avg. geodesic distance to the nearest wetland (m) [95% C.I.]^b^
First^c^	9/14 [64.29%]	14/14 [100%]	2655.73 [1595.67; 3715.78]
Second^c^	26/64 [40.63%]	46/64 [71.88%]	5058.46 [4089.39; 6027.52]

^a^As reported by the farm tenant.

^b^As calculated through Geographic Information System considering RAMSAR wetlands.

^c^Data were not reported in the epidemiological investigation forms of two IFs in the first epidemic wave and three IFs in the second wave; therefore the numbers of reference IFs are then n = 14 and n = 64 for the first and second epidemic waves respectively.

### Genetic analyses

**Figure 3:**
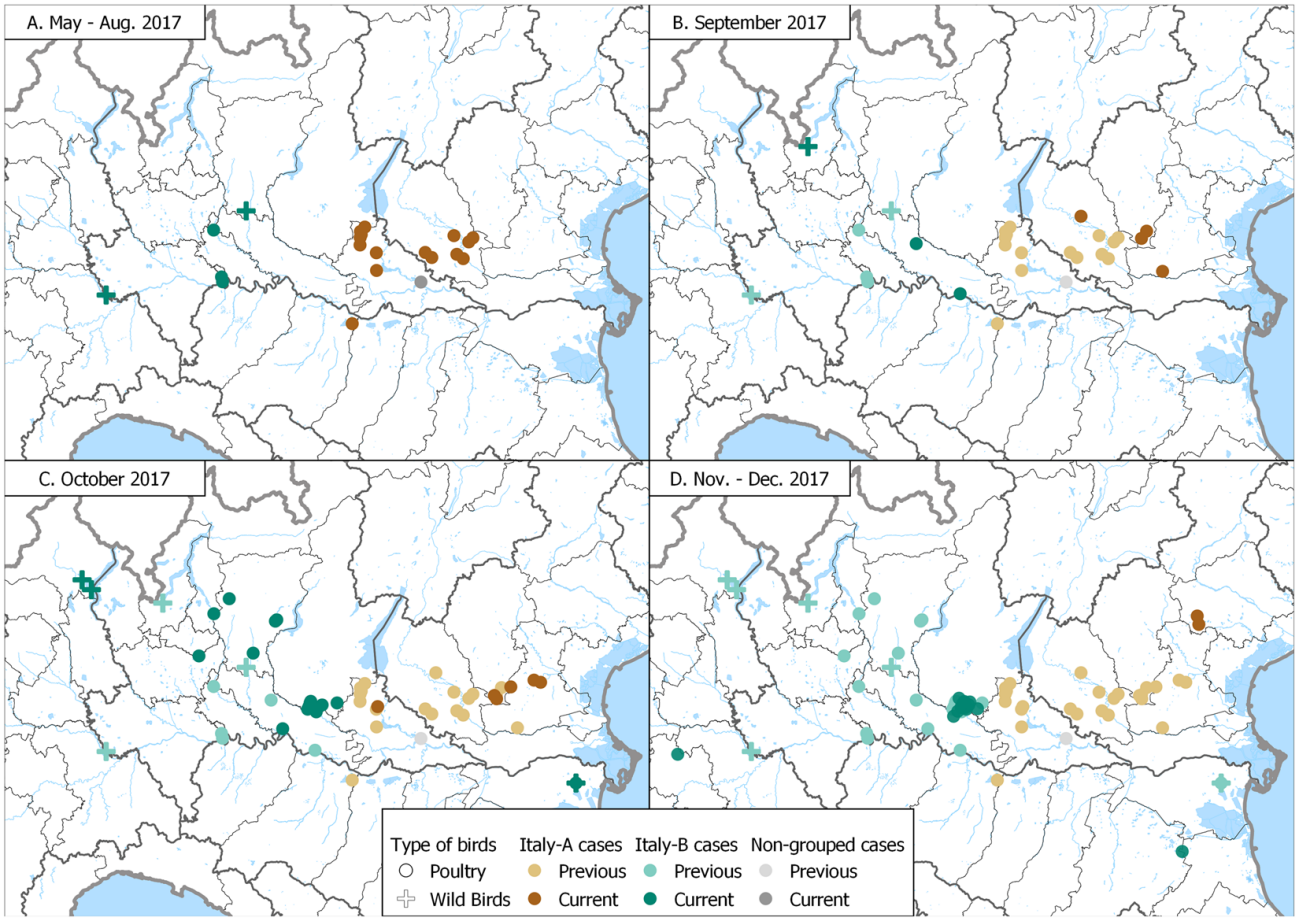
Distribution in space and time of cases classified into Italy-A and Italy-B subgroups; cases are classified as belonging to either the Italy-A or the Italy-B groups. ‘*Current cases’* refers to cases occurred in the period of reference indicated for each panel, ‘*Previous cases’* refers to cases occurred in preceding periods.

Maximum-likelihood phylogenetic trees of the eight gene segments indicated four distinct introductions of AIV genotypes at the beginning of the epidemic in Italy (December 2016-February 2017): H5N5 (2 cases in wild birds), H5N8-A/wild duck/Poland/82 A/2016-like, H5N8-A/painted stork/India/10CA03/2016-like, H5N8-A/mute swan/Croatia/70/2016-like (Supplementary Figs S1–S8)^12^. Except for H5N8-A/painted stork/India/10CA03/2016-like, which had been identified only in India, our phylogenetic analyses suggest that the other three genotypes were previously circulating in other European countries, with the H5N8-A/wild duck/Poland/82 A/2016-like resulting to be the most widespread. In particular, the Italian viruses showed the highest genetic relationship with H5N8 viruses collected in East Europe and Russia for all the gene segments (Supplementary Figs S1–S8). Since March 2017 one single genotype (H5N8-A/wild duck/Poland/82 A/2016-like) had been identified, with the exception of a single virus detected in a turkey farm in October, which resulted to be a reassortant virus for the NP and PA genes, likely acquired from low pathogenic viruses circulating in resident wild birds (Supplementary Figs S3 and S5). H5N8-A/wild duck/Poland/82 A/2016-like resulted to be the most prevalent genotype both wild and domestic birds during the first (60%) as well as the second (99%) epidemic wave. Topology of the eight phylogenies (Supplementary Figs S1–S8) indicates that over the second epidemic wave this genotype had further evolved into two main groups, named Italy-A and Italy-B, which seem to have circulated exclusively in the Italian territory. Specifically, Italy-B group can be clearly identified (bootstrap >60%) in all but the NS phylogeny, while Italy-A group shows high bootstrap supports (>60%) in the NP, PB1, PA and PB2 phylogenies. The first Italy-A group outbreak occurred in a rural farm in the eastern part of Lombardy region in late July (confirmation date: 20 July 2017), although later analyses indicated that also the last outbreak of the first wave was to be included in the Italy-A group. Virus belonging to this group circulated over the entire second epidemic wave exclusively in the north-east of Italy (Mantua province, Veneto region, and northern part of Emilia Romagna (Fig. 3). The last outbreaks due to Italy-A viruses were detected near the end of the second wave in two rural farms (confirmation dates: 23 November and 1 December 2017, respectively) in the east of Veneto region, after nearly two months from the previous detection (Fig. 3D). The Italy-B group was first detected in wild waterfowl in the western part of Lombardy region (confirmation date: 2 August 2017), then its occurrence was identified in geese and game bird farms in early-mid August. Italy-B viruses were detected in the industrial poultry sector since early September, eventually spreading to central-eastern Lombardy region, leading to the large cluster of cases in Brescia province (Fig. 3C,D).

**Figure 4:**
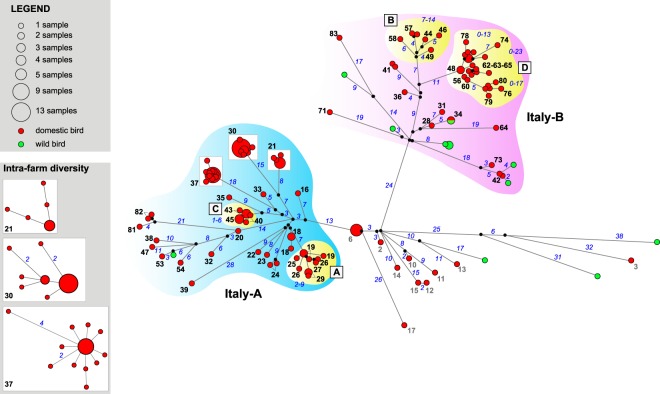
Median-joining phylogenetic network of the eight concatenated gene segments of the 2016-2017 Italian HPAI H5N8 viruses. Each unique sequence genotype is represented by a circle sized relatively to its frequency in the data set. Branches represent the shortest trees and are proportional to the number of nucleotide mutations (in blue) that separate each node. Median vectors are indicated as black circles.

Median-joining network analysis of the eight concatenated gene segments confirms the clustering of the Italian viruses of the second epidemic wave into the two groups (Fig. 4). During the second epidemic wave, 90% and 58% of the viruses detected in wild birds and domestic poultry respectively, belonged to the group Italy-B, while the remaining viruses were part of the group Italy-A.

To explore the within farm genetic variability, we sequenced multiple samples (from 2 to 21) collected from 18 infected farms at one or two sampling times (Supplementary Fig. S9). Surprisingly, we found a high intra-farm genetic variability, with viruses showing a number of nucleotide substitutions between zero and nine, dispersed throughout the genome. The gene codifying for the NS1 and NS2 proteins is the only gene segment showing no nucleotide changes at farm-level, while all the other gene segments show from 0 to 2 nucleotide substitutions (Supplementary Table S2). Considering the IFs from which we obtained at least three samples (10 farms), we calculated that the mean number of nucleotide substitutions in the genome per farm ranged from zero (cases 6 and 45) to five (case 19).

Analysis of the amino acid sequences showed the lack of known amino acid mutations associated to virus adaptation from wild to domestic birds, such as a shorter NA stalk or the presence of additional glycosylation sites in the HA protein^15^, in most of the Italian viruses. Only in a single virus belonging to the cluster of cases in Brescia province (IF no. 67) possessed a potential additional glycosylation site at position 215–217 (H5 numbering).

### Contact network assessment

**Figure 5:**
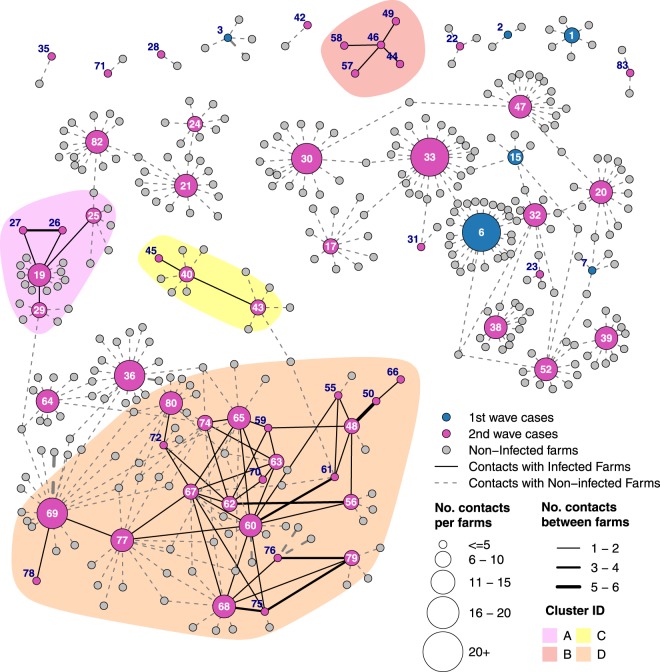
Representation of the network of contacts during for the 2017 H5N8 HPAI epidemic in Italy; numbers reported in the graph refer to the outbreak ID.

The information collected on contacts and genetic similarities between viruses were used to geographically visualise the network of contacts, and to assess its structure, revealing a remarkable interconnection between farms in the northern Italian regions (Figs 5 and S10). However, out of 482 at-contacts detected, only 70 (14.52%) of these occurred between IFs and could be inferred as potential sources for lateral spread (Table 4). All of the direct IF-to-IF connections were detected during the second epidemic wave. Movement of feed lorries was the most abundant information available (n = 314), although only nine contacts (2.87%) occurred directly between IFs. A limited number of IFs moved live poultry or their products (i.e. eggs) during their outbound risk period (ORP) (total number of movements = 10), and the proximity to roads where such vehicles went through was reported in the epidemiological investigations of two IFs (distances: 0.11 and 1.12 km respectively). The structure of the contact network indicated a high level of interconnectivity between IFs, although most of the contacts occurred via connections with non-infected farms (Fig. 5). In Fig. 5, only contacts occurring between IF in their ORP (i.e. period in which outbound contacts could potentially lead to infection in other farms) and IF in their inbound risk period (IRP, i.e. period in which inbound risk-contacts could have introduced the virus) were highlighted. Multiple contact types were observed between the 23 cases of the cluster identified in Lombardy region, with genetic closeness observed in many cases along with other contact types (Supplementary Fig. S11).

**Table 4:** Types of risk-contacts from infected farms (IFs); the number of connections from IF to IF is also reported indicating the proportion of potentially effective contacts.

Type of contacts	Total number	IF to IF contacts [%]	
Feed Lorries	314	9	[2.87%]
Owner/relatives	66	6	[9.09%]
Neighbourhood spread (1)	16	16	[100%]
Poultry company technician	18	0	—
Poultry company veterinarian	8	0	—
Proximity to roads	10	2	[20.00%]
Other tracked contacts	13	0	—
Untracked contacts (genomic evidence) (2)	37	37	[100]%
**Total**	**482**	**70**	**[14.52%]**

(1) Potential Neighbourhood spread was assessed only accounting the presence of other cases within 1500 meters from an Infected Farm (IF) in its outbound risk period (ORP).

(2) As genomic similarities were assumed as likely proxy for untracked contacts, this information was available only for Ifs.

**Figure 6:**
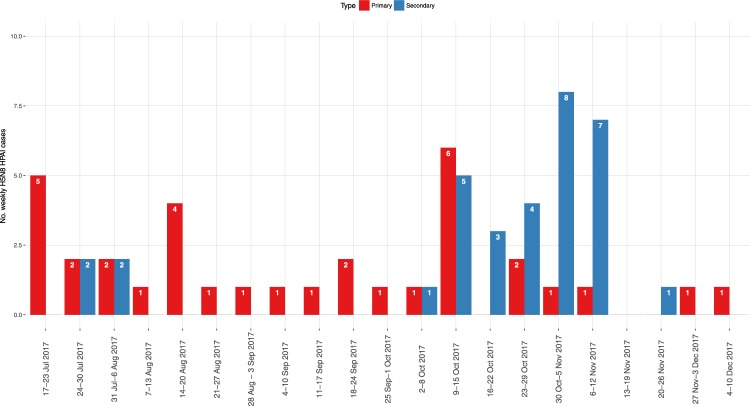
Second epidemic wave - Epidemic curve indicating the occurrence of primary (red) and secondary (blue) cases. The x-axis report date of symptoms onset.

Secondary cases were identified only in the second epidemic wave, including 32 IFs divided into 4 clusters (Supplementary Fig. S10). Viruses isolated from each of these clusters, grouped together in the MJ network (Fig. 4) and in the phylogenetic trees of all the eight AIV genes (Supplementary Figs S2–S9), supporting the findings of the epidemiological investigation. The large majority of secondary cases (28/32; 87.50%) were recorded from October 2017, reaching a peak between the last week of October and the first week of November, when the lateral spread occurrences matched and then exceeded the number of primary outbreaks (Fig. 6). When observing the proportion of the secondary cases on the total outbreaks occurred in the second wave, almost half of the IFs were related to lateral spread (32/67, 47.76%); the proportion increased up to 52.83% when considering cases in industrial poultry farms (28/53). The secondary cases of the large cluster in Lombardy region, accounted for about 80% of the total number of secondary outbreaks in the industrial sector (22/28, 78.57%), and represented a fraction of 41.51% of the total outbreaks detected in industrial poultry farms during the second epidemic wave (22/53). Areas where lateral spread was observed had an overall significantly greater farm density than areas where primary cases occurred in the second wave (F_(1,65)_ = 7.347, p < 0.01).

## Discussion

Starting on late December 2016, Italy had been affected by the circulation of HPAI H5 viruses previously reported in several other European Countries^2^. However, the epidemic that originated in Italy showed unique characteristics when compared with the viral spread reported almost elsewhere in Europe, such as the occurrence of a second wave of cases between July and December 2017. Although fattening turkeys were the most affected poultry production type, as observed in previous studies on HPAI and LPAI epidemics in Italy^11,16^, also backyard and layer farms were largely involved, and the virus was observed to spread to a number of chicken broiler farms starting on October. Backyard farms could have been affected due to lower overall biosecurity measures and higher numbers of untracked contacts; however, broilers had always been considered a low-risk type of poultry production^17^, and their involvement led to hypothesise a high level of viral pressure.

The two epidemic waves were characterised by different geographic distributions of cases. The limited number of IFs recorded in the first semester was likely due to the outbreaks not occurring in the core of the most densely populated poultry area of north-eastern Italy. Conversely, in the second epidemic wave virus introductions occurred into an area with one of the highest density of poultry farms in Europe^9^, with consequently higher probabilities of direct/indirect contacts between premises and lateral spread. In addition, the location of IFs in relation to presence of wetlands differed in the two waves. On average the cases in the second semester were situated significantly further away from water bodies than IFs in the first epidemic wave, suggesting different exposure to wild waterfowl and consequently different risk of AI virus introduction from wild birds to domestic poultry^18^.

The network of at-risk contacts between IFs and other farms revealed the high level of interconnection in the poultry sector. The integration of phylogenetic data with epidemiological information indicated that no IF-to-IF contacts occurred in the first epidemic wave; in fact, while most of the cases were included in the same network of contacts, all of the IFs were interconnected via contact-farms that did not become infected or did not result positive at repeated virological testing. On the contrary, four clusters of directly interconnected cases were observed in the second wave; in three of them lateral spread was inferable due to close proximities between cases, high genetic similarities, and/or trade of living poultry in live bird markets. The fourth and larger cluster included a series of cases located in a relatively small area, which occurred within five weeks and were the cause of the peaks in the epidemic curve during the second wave. A high number of connections related to proximity of cases and closeness to roads used to move infected birds, was also confirmed by genetic similarity, and indicated a potential high environmental contamination and spread of infection among poultry farms in the affected area.

The trend of primary and secondary cases indicated the passage from a phase with multiple point introductions of HPAI H5N8 from the wild reservoir and limited or no lateral spread of infection likely due to a lower density of poultry farms, to a more classical infectious disease epidemic in a densely populated livestock area. Nevertheless, only in one case point introductions from wild birds were followed by a rapid spread of the HPAI virus due to high-risk contacts between farms, leading to the large cluster in Brescia and Cremona provinces, suggesting the effectiveness of prevention and control measures in the remaining portions of the DPPA. Moreover, phylogenetic analyses revealed that, while the viruses detected between December 2016 and May 2017 belonged to neatly separated genetic groups within the clade 2.3.4.4-B, a single viral genotype became prevalent since March 2017^12^, and circulated throughout the second epidemic wave in both domestic poultry and wild birds. Nevertheless, in the second epidemic wave the virus further divided into two genomic groups (Italy-A and Italy-B), which circulated in distinct zones of northern Italy. The non-overlapping areas for the two identified groups were also likely related to the limited number of risk contacts between farms located in different regions, resulting from regionalisation measures applied for preventing the uncontrolled spread of AI. The presence of two spatially separated genomic groups, and their simultaneous eastward geographical shifting concurrently with the evolution of the epidemic, raised further questions on the role of wild waterfowl population ecology on the disease dynamics in the two areas, suggesting the presence of non-interacting meta-populations.

The detection of cases at the beginning of the second epidemic wave, in a period with low or no presence of migratory wild birds^19^, suggested that the virus might have spilled-over to the residential wild waterfowl populations. The finding of an infected juvenile grey heron, in close proximity to the last confirmed IF of the first wave in late May, also supported the hypothesis that the H5N8 HPAI virus likely spread to non-migratory wild birds. The virus could have amplified in the nestlings and juveniles^20^, and further dispersed in July – August, after leaving the areas where they gathered during the moulting period. Genetic analyses revealed the presence of a virus of the genotype H5N8-A/wild duck/Poland/82A/2016-like, in samples taken from the juvenile grey heron. The same genotype had been responsible for almost all the cases reported since May, providing further support to the hypothesis of spill-over to residential wild birds. This is also corroborated by the reassortant HPAI H5N8 virus found in a fattening turkey farm in October, indicating continuous H5N8 circulation in wild bird populations, together with other LP viruses. Wild birds infected with H5N8 viruses were also found in other European Countries until early-Autumn 2017, after the overall epidemic fading^1,21^.

Notwithstanding the role of migratory birds in the introduction of the H5N8 HPAI viruses in previously unaffected areas^2,13,22^, and the potential role of residential wild birds in amplifying the viral circulation, a very limited number of wild birds were found infected with AI viruses in Italy in 2016–2017. Furthermore, also non-waterfowl tested positive during the second wave in proximity of a large infected layer premise, indicating a likely high viral pressure in the environment at the domestic poultry/wild birds interface. The scarce number of cases in wild birds in Italy could have been due to a number of factors. Firstly, the disease dynamics in north Italy might have differed from what observed in other European Countries. Although this could be feasible, the scarce information available made this hypothesis hardly verifiable. Secondly, other productive types such as backyard/familiar farms could have played an important role in the viral maintenance and/or amplification. Nevertheless, the lack of aminoacid mutations associated to adaptation to domestic birds, in association with no evidence of HPAI virus circulation revealed by intensive monitoring activities in such farming types, seemed to rule out the latter hypothesis. Thirdly, and most likely, the limited efficacy of AI surveillance on wild birds on the Italian territory could have led to an underestimation of the H5N8 HPAI virus presence. In fact, until 2017 only passive surveillance measures had been applied with not completely defined procedures to alert and notify the presence of dead/symptomatic birds to the competent authorities. This also prompted to the need of outlining a more structured surveillance plan on wild birds, which accounts also for the migratory flows and the ecology of the different waterfowl populations.

The integration of epidemiological evidences with genetic analyses helped in examine in detail the dynamics of H5N8 HPAI in Italy, providing valuable knowledge on the occurrence of lateral spread and hence indicating areas eligible for targeted control measures and enhanced biosecurity. This type of analyses could be performed in almost a real-time fashion, generating quickly accessible and easy to explain pieces of information to generate and/or corroborate hypotheses on the more likely epidemiologic pattern of contacts between cases, in order to adapt AI eradication and prevention policies according to the changes in the epidemiological pattern of the disease. Nevertheless, several aspects of the dynamics of the spread of the 2016-2017 H5N8 HPAI epidemic from wild birds to domestic poultry are still not completely clear. While breaches in biosecurity measures were detected in some of the reported outbreaks, cases occurred also in farms with high biosecurity statuses as observed in other EU Countries^23^. This prompted a preliminary assessment of biosecurity at farm-level in the most affected regions, granting permission to house birds only to premises that fully comply with the well-defined standards.

## Methods

### Epidemiological Investigation

As provided by the European legislation on Avian Influenza control^24,25^, a thorough epidemiological inquiry was carried out in all of the IFs by means of a standardized questionnaire, to collect information on how the virus could have been introduced, and whether further spread to other poultry farms was likely. The collected information included:

(i) managerial and demographical aspects of the farm, including type of sheds, species reared, productive type, number of poultry and housing date(s);
(ii) onset and type of observed symptoms referred by the farmer/private practitioner, the daily mortality was also collected for each farm, and when possible for each shed;
(iii) number of poultry farms, and productive types, within the Protection and Surveillance Zones of the IF (i.e. 3 and 10 km radius respectively, as indicated in the applicable legislation)^24^;
(iv) movements of people, live poultry, poultry products (e.g. eggs), materials (e.g. manure), equipment and vehicles, according to the movements and visits registry kept in the farm;
(v) presence of wild birds, as reported by the farmer;
(vi) presence of water bodies in proximity to the affected premises, as reported by the farmer.

The number of farms within a 10-km radius from the IFs was considered as a measure of the overall farm density around the outbreak. Furthermore, information on presence of wetlands were also derived through scanning remote sensed images^26^, and geodesic distances from farms to the nearest wetland were calculated as a proxy of the risk to come into contact with wild waterfowl^27^.

### High-throughput sequencing and data analysis

The complete genomes of a total 172 H5N8/H5N5 positive samples, including 10 samples from previous studies^12^, were sequenced to explore the inter- and intra-farm genetic variability; 155 samples were collected from the 83 IFs, and 17 from wild birds (Supplementary Fig. S1).

RNA was purified using the Nucleospin RNA kit (Macherey–Nagel, Duren, Germany) from clinical samples (tissues or swabs). Complete influenza A virus genomes were amplified with the SuperScript III One-Step RT-PCR system with Platinum Taq High Fidelity (Invitrogen, Carlsbad, CA) using one pair of primers complementary to the conserved elements of the influenza A virus promoter (MBTUni-12-DEG 5′-GCGTGATCAGCRAAAGCAGG-3′ and MBTUni-13 5′-ACGCGTGATCAGTAGAAACAAGG-3′)^28^. Sequencing libraries were obtained using Nextera DNA XT Sample preparation kit (Illumina), and quantified using the Qubit dsDNA High Sensitivity kit (Invitrogen, USA). The average fragment length was determined using the Agilent High Sensitivity Bioanalyzer Kit. The indexed libraries were pooled in equimolar concentrations and sequenced on Illumina MiSeq.

Illumina reads quality was assessed using FastQC v0.11.2; raw data were filtered by removing:

(i) reads with more than 10% of undetermined (“N”) bases;
(ii) reads with more than 100 bases with Q score below 7;
(iii) duplicated paired-end reads.

Remaining reads were clipped from Illumina Nextera XT adaptors using scythe v0.991 (https://github.com/vsbuffalo/scythe) and trimmed with sickle v1.33 (https://github.com/najoshi/sickle). Reads shorter than 80 bases or unpaired after previous filters were discarded. High quality reads were aligned against a reference genome using BWA v0.7.12^29^, obtaining an average sequence coverage of 27,000. Alignments were processed with Picard-tools v2.1.0 (http://picard.sourceforge.net) and GATK v3.5^30–32^, to correct potential errors, realign reads around indels and recalibrate base quality. Single Nucleotide Polymorphisms (SNPs) were called using LoFreq v2.1.2^33^, and the outputs were used to generate consensus sequences.

All AI viruses were handled according to the OIE requirements for bio-safety and bio-containment^34,35^.

### Phylogenetic analyses

Consensus sequences of the complete genome of a single virus for IF were aligned using MAFFT v. 7^36^, and compared to the most related sequences available in GISAID. Maximum likelihood (ML) phylogenetic trees were obtained for each gene segment using the best-fit general time-reversible (GTR) model of nucleotide substitution, with gamma-distributed rate variation among sites (with four rate categories, Γ4), and a heuristic SPR branch-swapping search available in the PhyML program version 3.1^37^. One thousand bootstrap replicates were performed to assess the robustness of individual nodes of the phylogeny. Phylogenetic trees were visualized with the program FigTree v,1.4.2 (http://tree.bio.ed.ac.uk/software/figtree/).

The eight concatenated gene segments of 163 HPAI H5N8 influenza viruses belonging to the Poland-like genetic group were tested for reassortment using the GENECOV, Bootscan, MaxChi, RDP, SiScan methods available in the RDP4 program^38^. Phylogenetic networks were constructed using the Median Joining (MJ) method implemented in the program NETWORK 4.6.1.6^39^, for the concatenated gene segments of non-reassortant viruses of the Poland-like group as well as of each of the four clusters of directly interconnected cases. This method uses a parsimony approach to reconstruct the relationships between highly similar sequences, and allows the creation of *median vectors*, which represents un-sampled sequences used to connect the existing genotypes in the most parsimonious way. The parameter epsilon was set to zero, to minimise the number of median vectors.

### Contact network

In order to hypothesise whether the AI virus could have spread between farms, the following dates of interest were calculated for each IF:

(i) potential introduction of influenza viruses, calculated considering a maximum incubation period of two weeks (14 days) from symptoms onset^40^;
(ii) onset of symptoms, calculated considering the information collected during the epidemiological inquiry, or the start of increased mortality, whichever came earlier;
(iii) date of suspicion, corresponding to the date when the outbreak was reported to the local veterinary authorities;
(iv) date of confirmation, the day when the laboratory tests indicated the presence of an HPAI H5N8 virus;
(v) outbreak extinction date, which was considered being occurred after culling of poultry in the infected farm and disposal of carcasses, and followed by the application of preliminary cleansing and disinfection measures.

The period in which AI viruses could have spread from each IF was assumed as starting from 2 days before the onset of symptoms, and ending with the extinction and preliminary cleansing and disinfection of the outbreak site^41^ (outbound risk period – ORP). Conversely, each IF was considered having potentially been infected in a period between the estimated date of AI virus introduction, and the onset of symptoms or the beginning of the increase in the mortality rate minus 2 days, assumed as minimum incubation period for HPAI^16^ (inbound risk period – IRP).

The network of contacts was investigated by considering both the information collected through the epidemiological investigation and the results of the genomic analyses. Potential contacts were classified into:

(i) belonging to same owner, or close relatives (to a previously detected IF);
(ii) movement of feed lorries;
(iii) contacts through personnel operating for the same poultry company (i.e. technicians and veterinarians);
(iv) proximity to IFs in ORP (i.e. within 1500 m), indicating the chance of neighbourhood spread occurring by non-recorded means (e.g. environmental contamination, or aerosol spread)^41,42^;
(v) indirect contacts via contaminated vehicles, due to closeness to roads used for moving live poultry and/or their products from IFs in the ORP;
(vi) other contacts, encompassing less frequent contact types (e.g. vehicles to collect and transport eggs, lorries for transporting bedding materials, and vehicles for the removal of carcasses);
(vii) results of the MJ network, assumed to indicate potential (untracked) contacts.

The directionality of contacts was taken into account for risk contacts occurring via movements of vehicles and/or personnel. Contacts between IFs were only accounted for when occurring within both the ORP of the originating IF, and the IRP of the receiving IF. Farms belonging to the same owner, contacts related to neighbourhood spread, and/or genetically similar viruses were considered as *non-directional* contacts, as it was not possible to infer the most likely origin of the spread.

An IF was defined as *primary case* when no at-risk contacts were identified with previously detected outbreak, and no phylogenetic evidence emerged that the virus was closely related to viruses already isolated from poultry in the affected area. Conversely, when at-risk inbound contacts could be reliably traced back to an IF in ORP, and/or genomic analyses provided strong support, an outbreak was defined as *secondary case*.

### Experiments on animals/humans

The study did not include any experiment on animals and/or humans. Samples were taken from dead birds in infected farms, as part of the measures provided by the European Council Directive on Avian Influenza (Council Directive 2005/94/EC)^24^.

## Supplementary information

Supplementary Materials

## Data Availability

Complete genome sequences of one representative virus from each IF generated in this study were deposited in the GISAID EpiFlu Database (https://platform.gisaid.org/) under accession numbers: EPI1261344- EPI1261994, EPI961493-EPI961507, EPI961509-EPI961522, EPI961524-EPI961527, EPI1040223-EPI1040238, EPI1081918-EPI1081924, EPI1081966-EPI1081973. Data on characteristics of outbreaks (ID, geographical location, productive type, dates of symptoms onset, confirmation, and outbreak extinction) are available at the Supplementary Table S1. More detailed information on contacts between farms is available upon request to the corresponding author.
