# Supplementary material for: Integration of genetic and epidemiological data to infer H5N8 HPAI virus transmission dynamics during the 2016-2017 epidemic in Italy
Source: Sci Rep. 2018 Dec 21;8:18037. doi: 10.1038/s41598-018-36892-1 (PMC6303474; doi:10.1038/s41598-018-36892-1)
Supplement: Supplementary file 1 — Supplementary Materials [file 41598_2018_36892_MOESM1_ESM.pdf]

Integration of genetic and epidemiological data to infer H5N8 HPAI virus transmission dynamics during the 2016-2017 epidemic in Italy

Mulatti, P.*, Fusaro, A., Scolamacchia, F., Zecchin, B., Azzolini, A., Zamperin, G., Terregino, C., Cunial, G., Monne, I., Marangon, S.

Istituto Zooprofilattico Sperimentale delle Venezie, Legnaro (Padua), Italy

* Corresponding Author

Supplementary Figure S1. Maximum Likelihood phylogenetic tree of the PB2 gene. The 2016-2017 Italian HPAI H5N8 isolated from poultry are marked in red. The 2016-2017 Italian HPAI H5N8 isolated from wild birds are marked in green. The light blue box shows the Italy A group; the violet box shows the Italy B group. Epidemiological clusters are marked with a yellow bar on the side. Bootstrap supports higher than 600/1000 are indicated next to the nodes; branch lengths are scaled according to the numbers of nucleotide substitutions per site.

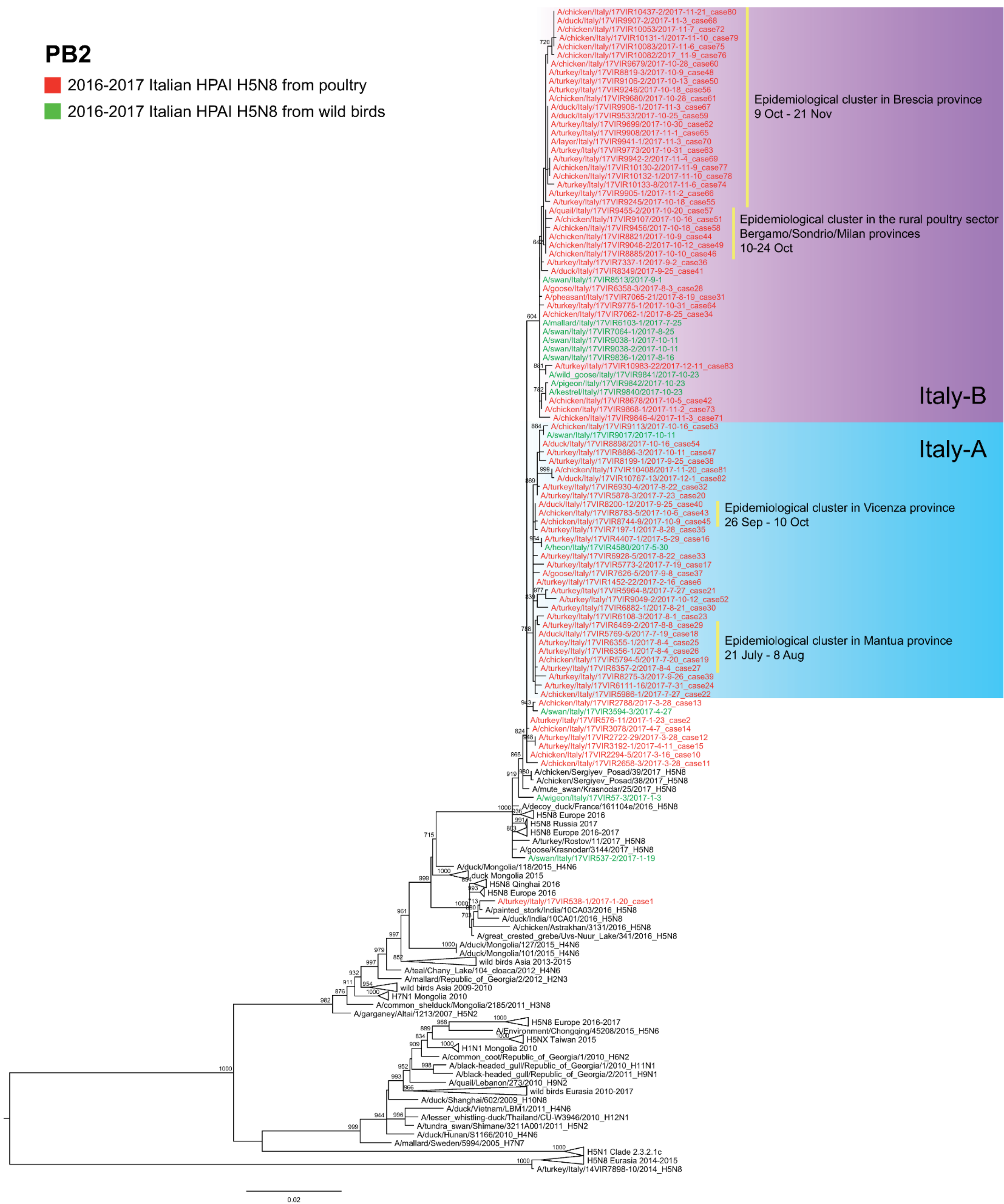

Supplementary Figure S2. Maximum Likelihood phylogenetic tree of the PB1 gene. The 2016-2017 Italian HPAI H5N8 isolated from poultry are marked in red. The 2016-2017 Italian HPAI H5N8 isolated from wild birds are marked in green. The light blue box shows the Italy A group; the violet box shows the Italy B group. Epidemiological clusters are marked with a yellow bar on the side. Bootstrap supports higher than 600/1000 are indicated next to the nodes; branch lengths are scaled according to the numbers of nucleotide substitutions per site.

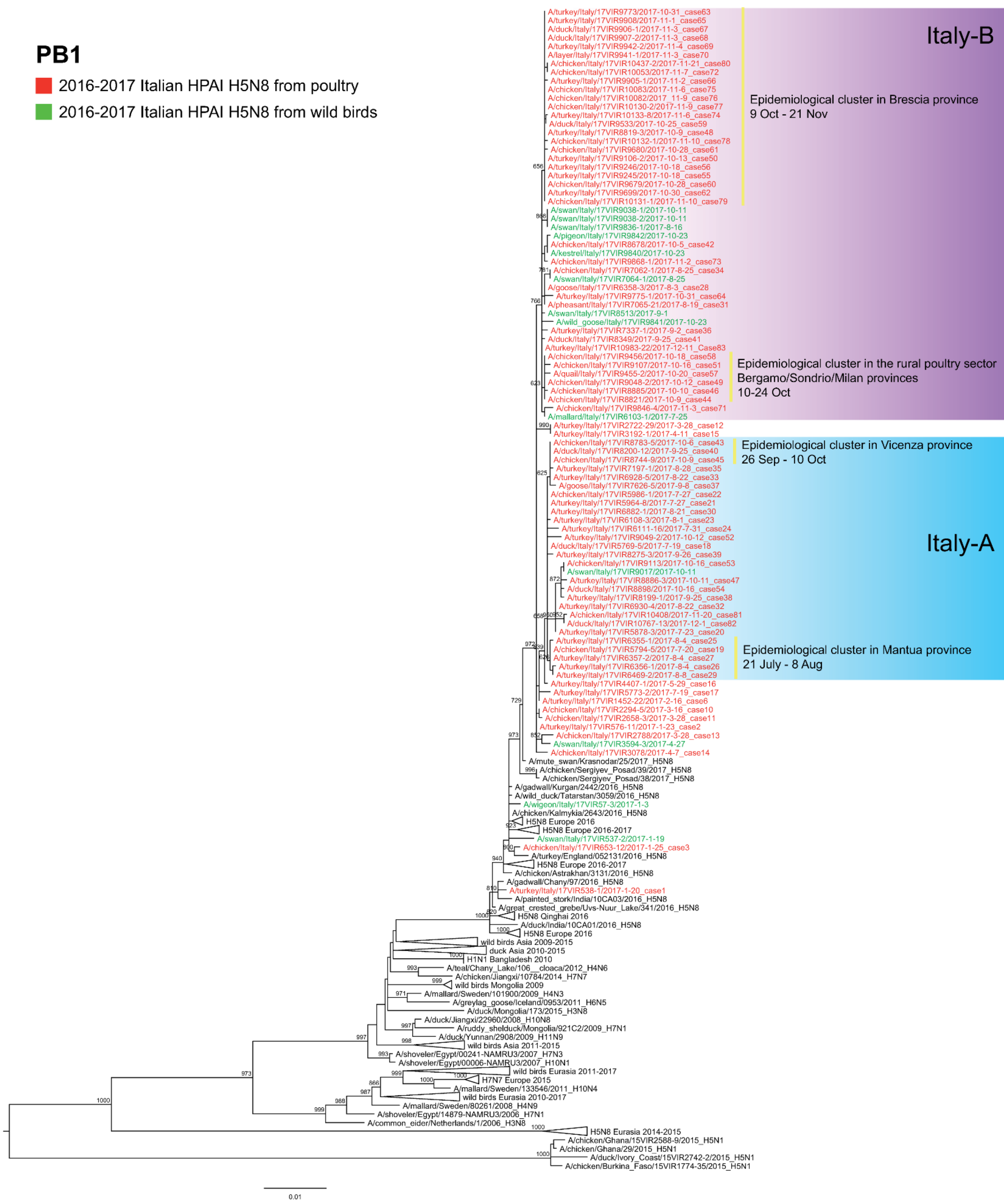

Supplementary Figure S3. Maximum Likelihood phylogenetic tree of the PA gene. The 2016-2017 Italian HPAI H5N8 isolated from poultry are marked in red. The 2016-2017 Italian HPAI H5N8 isolated from wild birds are marked in green. The light blue box shows the Italy A group; the violet box shows the Italy B group. Epidemiological clusters are marked with a yellow bar on the side. Bootstrap supports higher than 600/1000 are indicated next to the nodes; branch lengths are scaled according to the numbers of nucleotide substitutions per site.

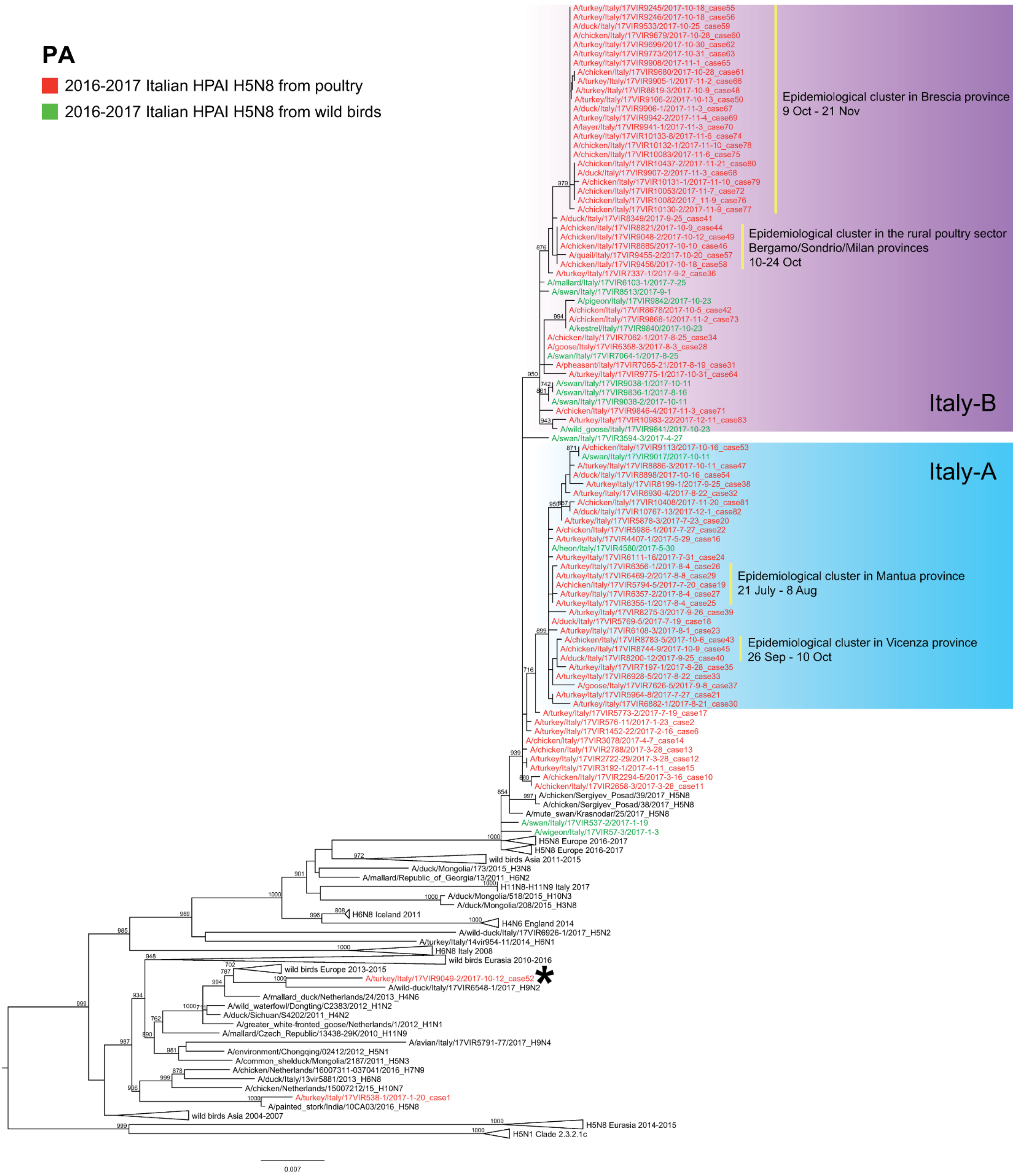

Supplementary Figure S4. Maximum Likelihood phylogenetic tree of the HA gene. The 2016-2017 Italian HPAI H5N8 isolated from poultry are marked in red. The 2016-2017 Italian HPAI H5N8 isolated from wild birds are marked in green. The light blue box shows the Italy A group; the violet box shows the Italy B group. Epidemiological clusters are marked with a yellow bar on the side. Bootstrap supports higher than 600/1000 are indicated next to the nodes; branch lengths are scaled according to the numbers of nucleotide substitutions per site.

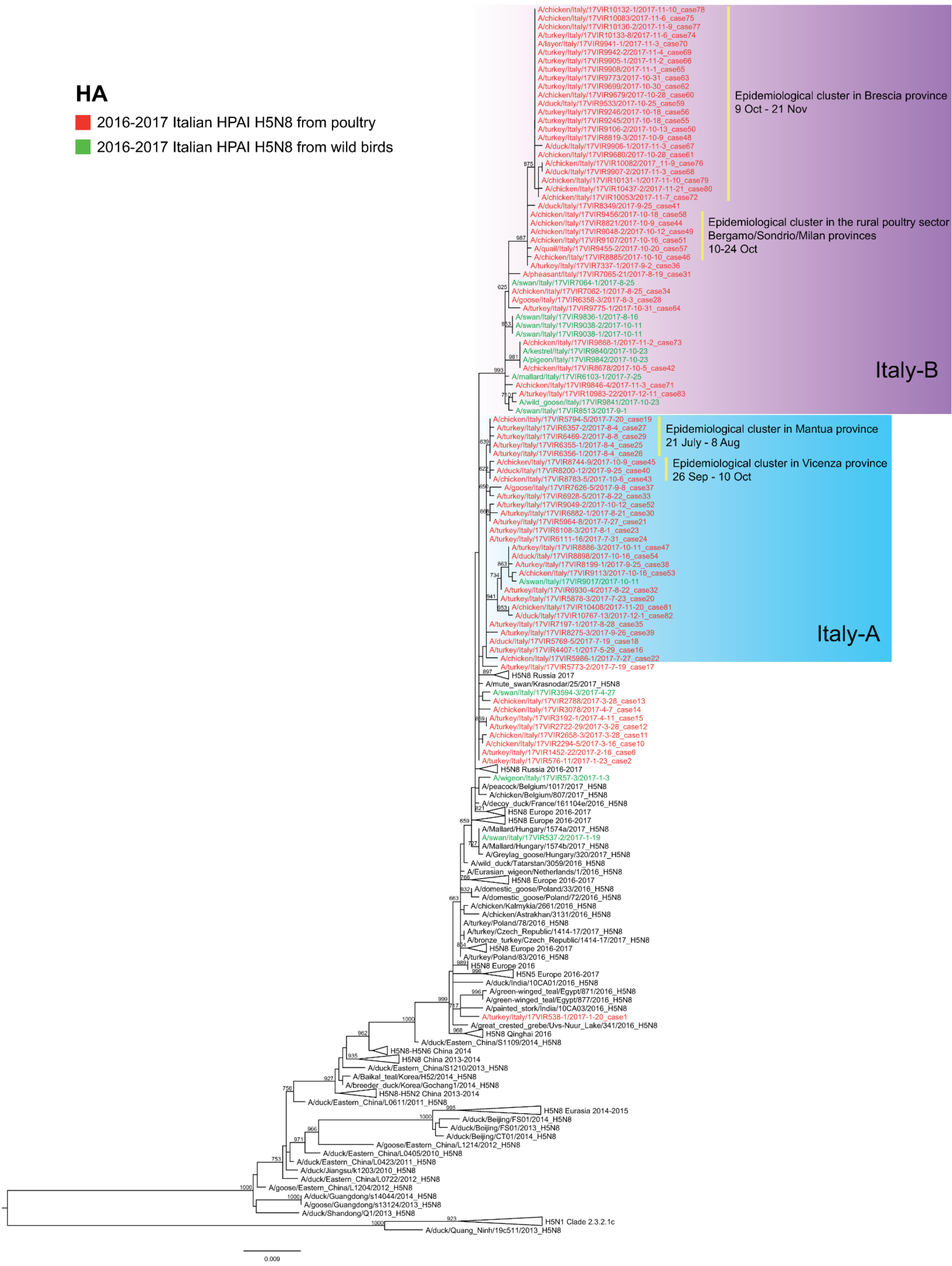

Supplementary Figure S5. Maximum Likelihood phylogenetic tree of the NP gene. The 2016-2017 Italian HPAI H5N8 isolated from poultry are marked in red. The 2016-2017 Italian HPAI H5N8 isolated from wild birds are marked in green. The light blue box shows the Italy A group; the violet box shows the Italy B group. Epidemiological clusters are marked with a yellow bar on the side. Bootstrap supports higher than 600/1000 are indicated next to the nodes; branch lengths are scaled according to the numbers of nucleotide substitutions per site.

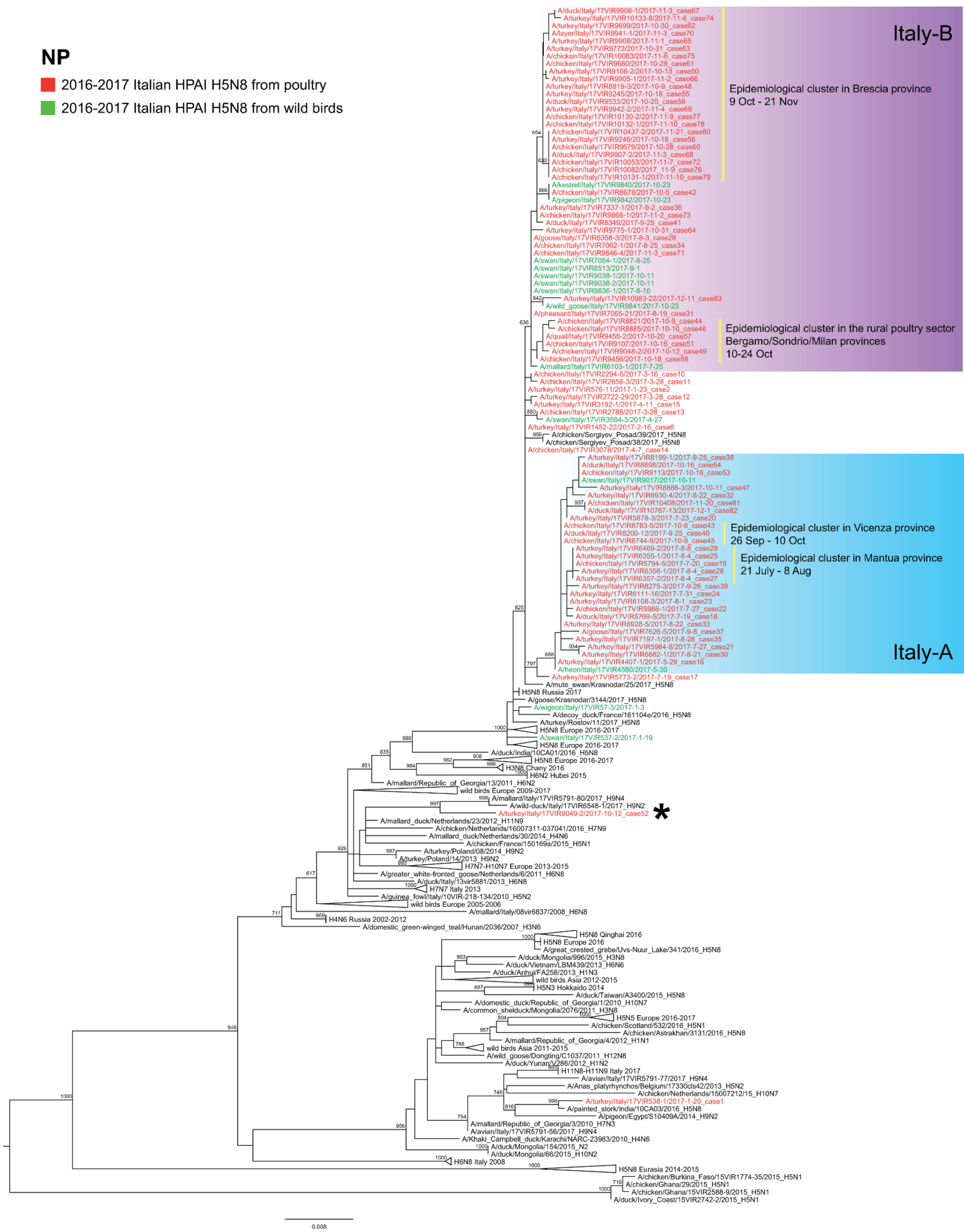

Supplementary Figure S6. Maximum Likelihood phylogenetic tree of the NA gene. The 2016-2017 Italian HPAI H5N8 isolated from poultry are marked in red. The 2016-2017 Italian HPAI H5N8 isolated from wild birds are marked in green. The light blue box shows the Italy A group; the violet box shows the Italy B group. Epidemiological clusters are marked with a yellow bar on the side. Bootstrap supports higher than 600/1000 are indicated next to the nodes; branch lengths are scaled according to the numbers of nucleotide substitutions per site.

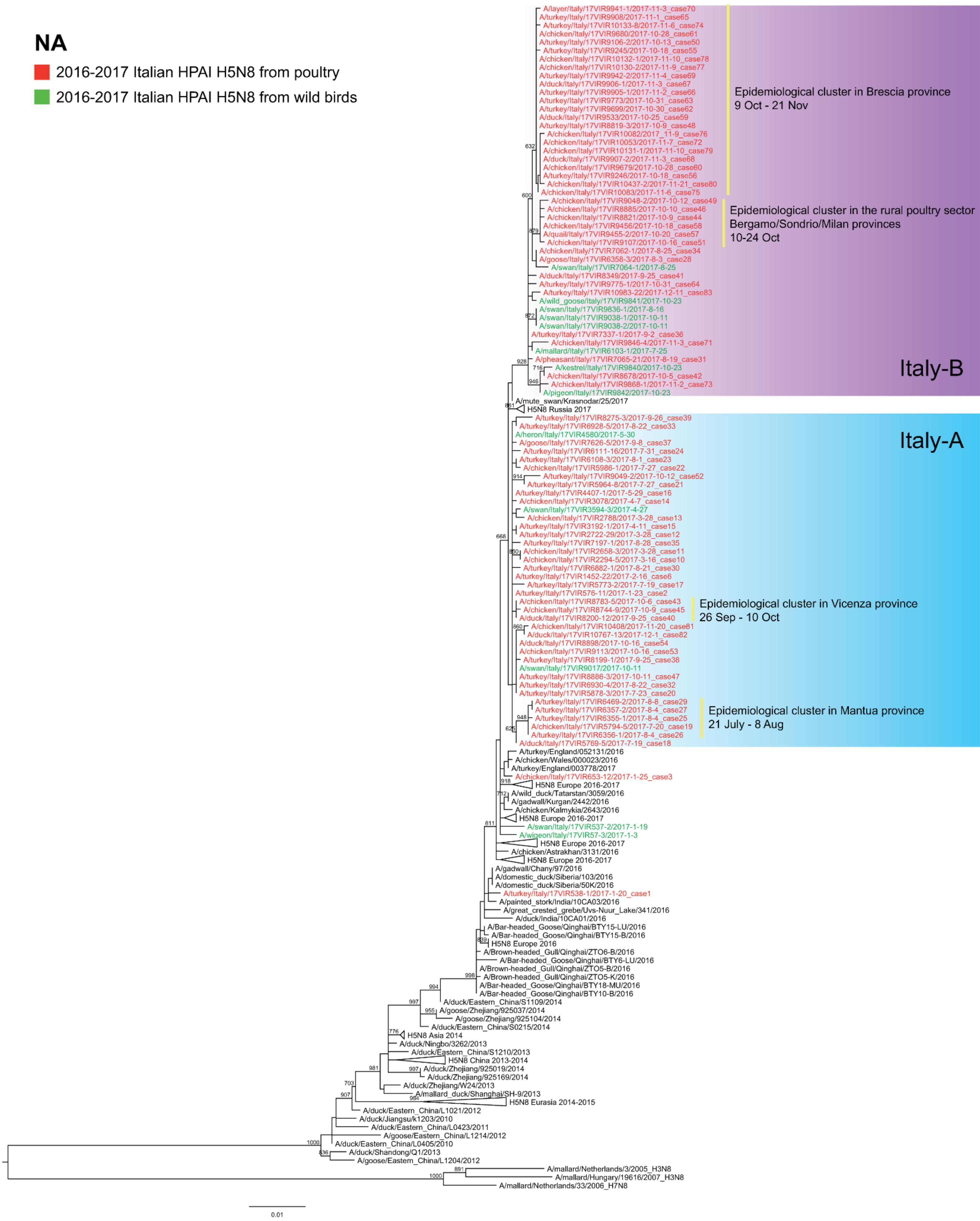

Supplementary Figure S7. Maximum Likelihood phylogenetic tree of the MP gene. The 2016-2017 Italian HPAI H5N8 isolated from poultry are marked in red. The 2016-2017 Italian HPAI H5N8 isolated from wild birds are marked in green. The light blue box shows the Italy A group; the violet box shows the Italy B group. Epidemiological clusters are marked with a yellow bar on the side. Bootstrap supports higher than 600/1000 are indicated next to the nodes; branch lengths are scaled according to the numbers of nucleotide substitutions per site.

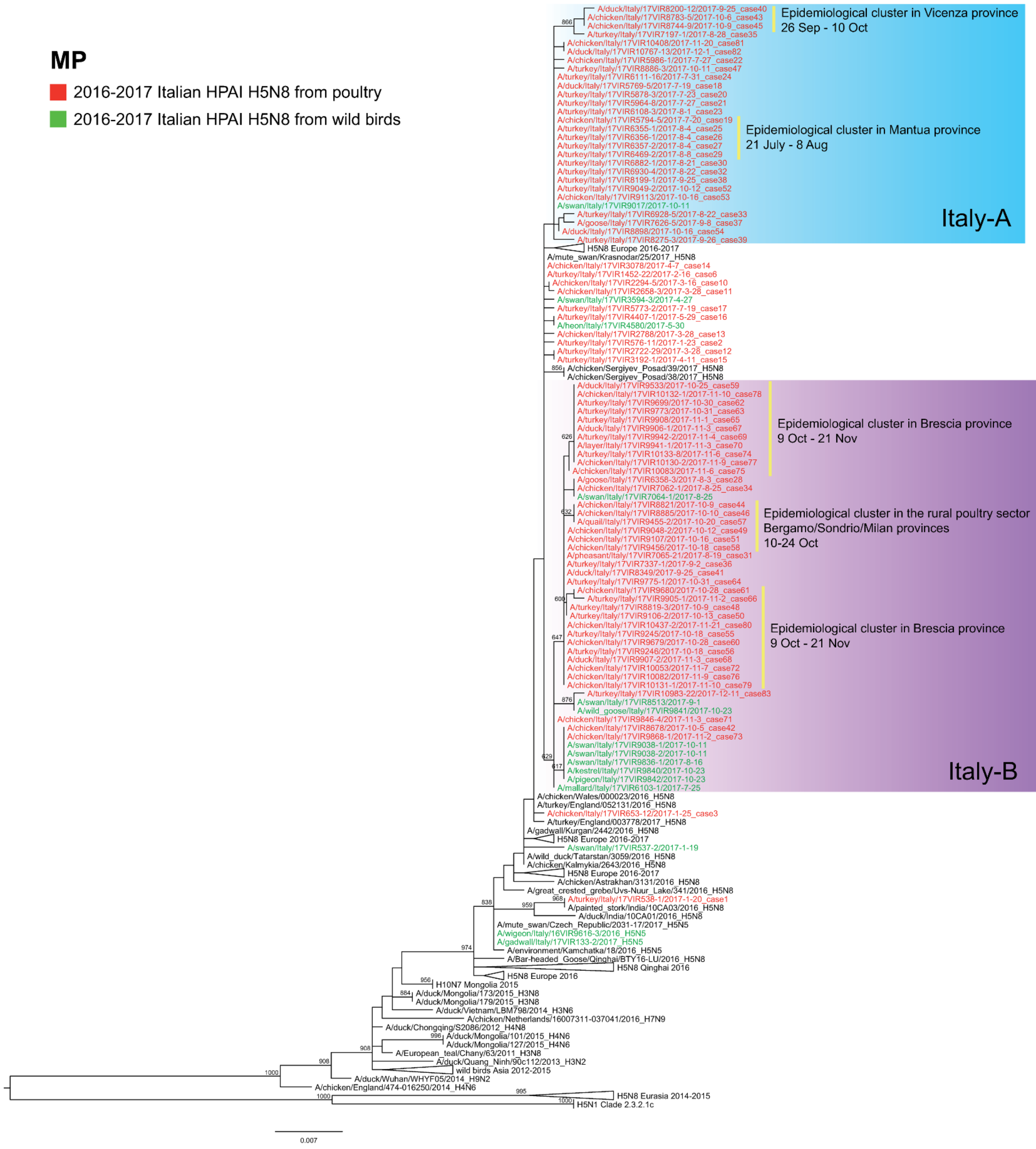

Supplementary Figure S8. Maximum Likelihood phylogenetic tree of the NS gene. The 2016-2017 Italian HPAI H5N8 isolated from poultry are marked in red. The 2016-2017 Italian HPAI H5N8 isolated from wild birds are marked in green. Epidemiological clusters are marked with a yellow bar on the side. Bootstrap supports higher than 600/1000 are indicated next to the nodes; branch lengths are scaled according to the numbers of nucleotide substitutions per site.

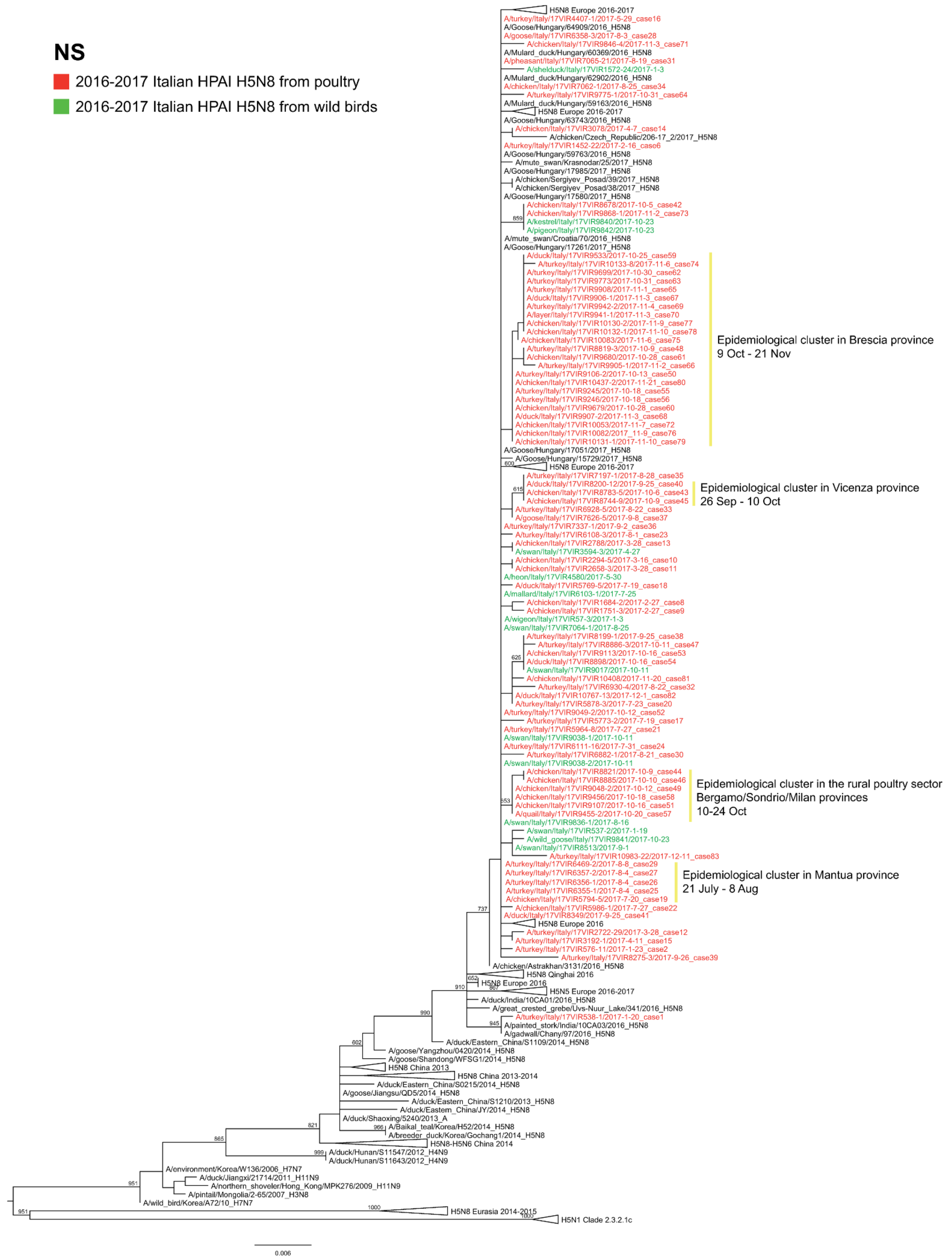

Supplementary Figure S9. H5N8 cases in domestic poultry in Italy in 2016-2017: number of sequenced viruses for each infected farm.

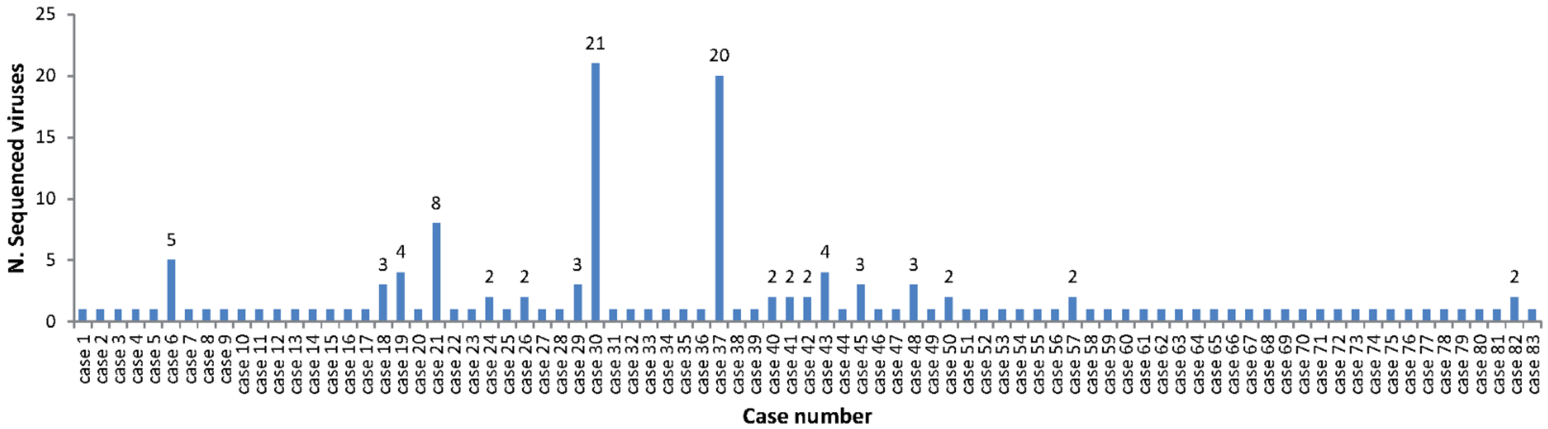

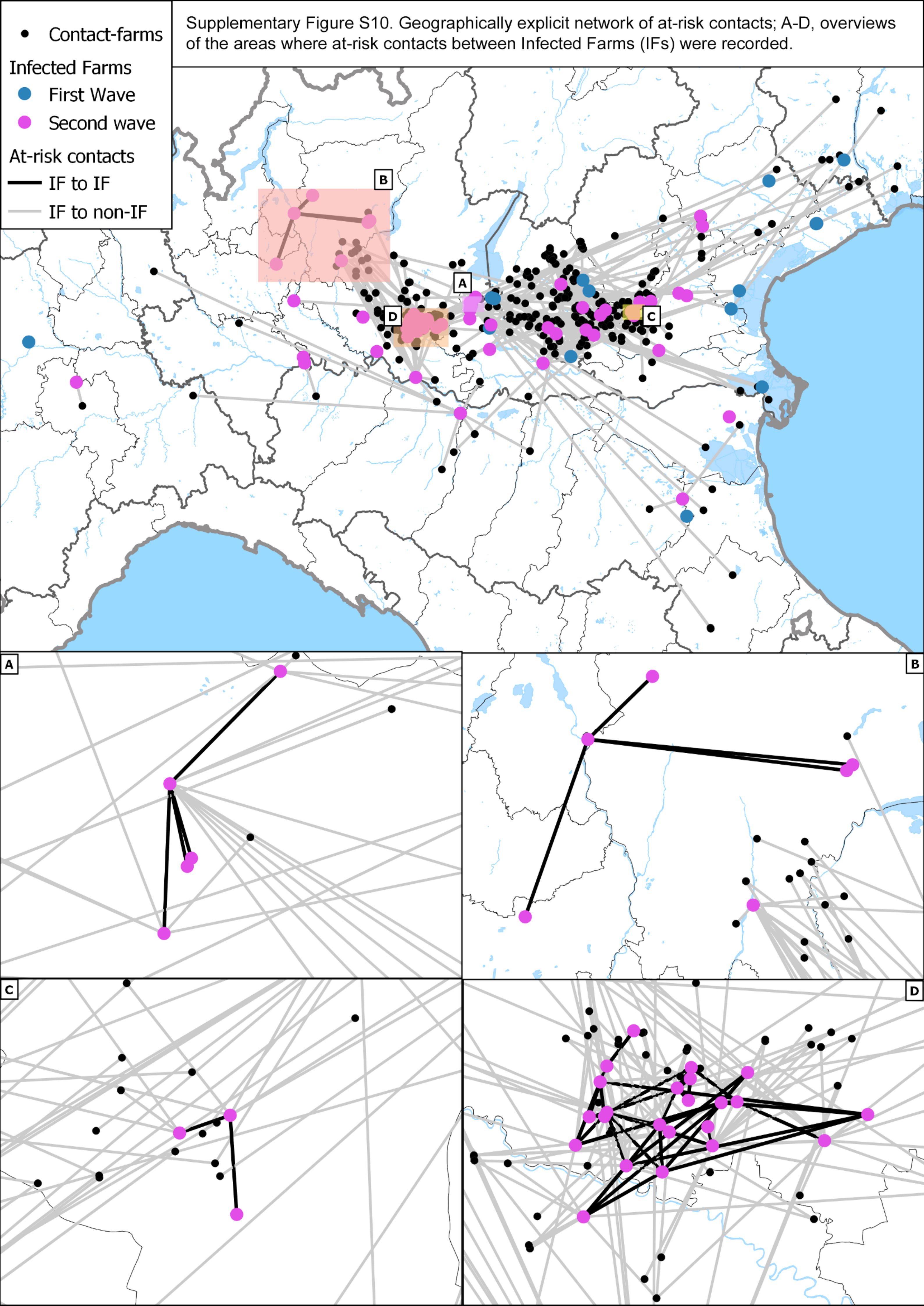

Supplementary Figure S10. Geographically explicit network of at-risk contacts; A-D, overviews of the areas where at-risk contacts between Infected Farms (IFs) were recorded.

Supplementary Figure S11. Details of the Infected farm-to-Infected farm contacts in the Brescia cluster (Cluster D in figures 5 and Supplementary figure S10).

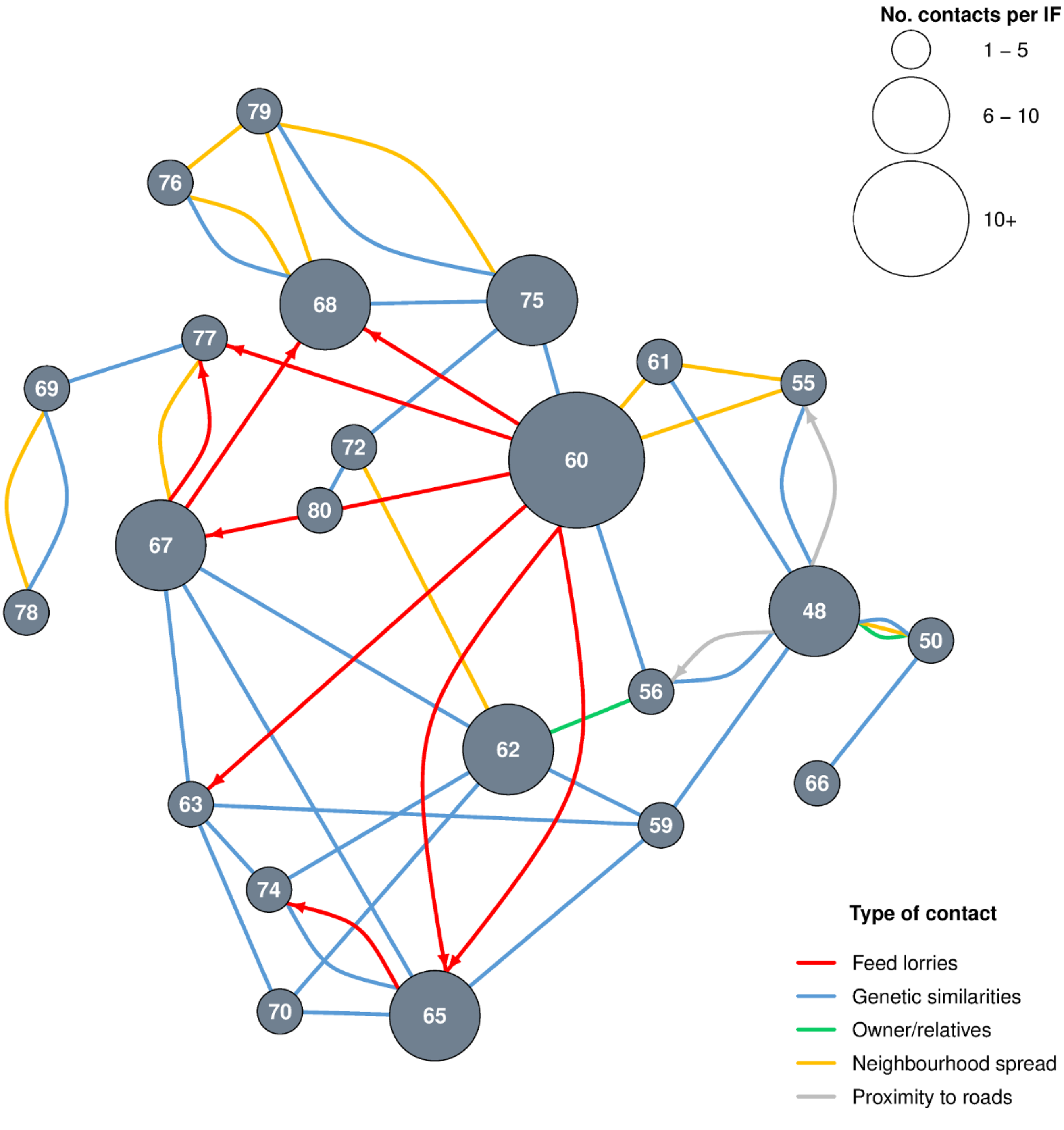

Supplementary Table S1. Main characteristics of the outbreaks in domestic poultry detected during the 2016-2017 H5N8 HPAI epidemic in Italy.

ID	Wave	Type (Primary/Secondary)	Region	Province	Productive Type	Symptoms Onset	Confirmation Date	Extinction Date
1	1	Primary	Veneto	Venice	Fattening Turkeys	17-Jan-2017	21-Jan-2017	26-Jan-2017
2	1	Primary	Veneto	Padua	Fattening Turkeys	23-Jan-2017	23-Jan-2017	29-Jan-2017
3	1	Primary	Veneto	Rovigo	Laying Hens	23-Jan-2017	25-Jan-2017	4-Feb-2017
4	1	Primary	Emilia Romagna	Parma	Fattening Turkeys	1-Feb-2017	2-Feb-2017	9-Feb-2017
5	1	Primary	Lombardy	Mantua	Fattening Turkeys	13-Feb-2017	15-Feb-2017	18-Feb-2017
6	1	Primary	Veneto	Verona	Fattening Turkeys	16-Feb-2017	17-Feb-2017	21-Feb-2017
7	1	Primary	Lombardy	Mantua	Fattening Turkeys	21-Feb-2017	22-Feb-2017	25-Feb-2017
8	1	Primary	Veneto	Venice	Backyard	27-Feb-2017	28-Feb-2017	28-Feb-2017
9	1	Primary	Veneto	Venice	Backyard	27-Feb-2017	28-Feb-2017	1-Mar-2017
10	1	Primary	Veneto	Treviso	Backyard	16-Mar-2017	16-Mar-2017	18-Mar-2017
11	1	Primary	Veneto	Verona	Fattening Turkeys	27-Mar-2017	29-Mar-2017	31-Mar-2017
12	1	Primary	Friuli Venezia Giulia	Pordenone	Backyard	28-Mar-2017	28-Mar-2017	30-Mar-2017
13	1	Primary	Piedmont	Turin	Backyard	20-Mar-2017	30-Mar-2017	30-Mar-2017
14	1	Primary	Emilia Romagna	Bologna	Laying Hens	5-Apr-2017	8-Apr-2017	17-Apr-2017
15	1	Primary	Veneto	Verona	Fattening Turkeys	9-Apr-2017	12-Apr-2017	12-Apr-2017
16	1	Primary	Lombardy	Mantua	Fattening Turkeys	27-May-2017	30-May-2017	4-Jun-2017
17	2	Primary	Lombardy	Mantua	Fattening Turkeys	17-Jul-2017	20-Jul-2017	23-Jul-2017
18	2	Primary	Lombardy	Mantua	Backyard	17-Jul-2017	20-Jul-2017	20-Jul-2017
19	2	Primary	Lombardy	Mantua	Laying Hens	19-Jul-2017	21-Jul-2017	6-Aug-2017
20	2	Primary	Veneto	Verona	Fattening Turkeys	23-Jul-2017	25-Jul-2017	27-Jul-2017
21	2	Primary	Veneto	Verona	Fattening Turkeys	23-Jul-2017	27-Jul-2017	30-Jul-2017
22	2	Primary	Lombardy	Mantua	Laying Hens	27-Jul-2017	27-Jul-2017	7-Aug-2017
23	2	Primary	Veneto	Verona	Fattening Turkeys	28-Jul-2017	2-Aug-2017	2-Aug-2017
24	2	Primary	Emilia Romagna	Parma	Fattening Turkeys	31-Jul-2017	2-Aug-2017	8-Aug-2017
25	2	Secondary	Lombardy	Mantua	Fattening Turkeys	4-Aug-2017	5-Aug-2017	11-Aug-2017
26	2	Secondary	Lombardy	Mantua	Fattening Turkeys	29-Jul-2017	5-Aug-2017	11-Aug-2017

ID	Wave	Type (Primary/Secondary)	Region	Province	Productive Type	Symptoms Onset	Confirmation Date	Extinction Date
27	2	Secondary	Lombardy	Mantua	Fattening Turkeys	28-Jul-2017	5-Aug-2017	11-Aug-2017
28	2	Primary	Lombardy	Pavia	Geese	3-Aug-2017	5-Aug-2017	8-Aug-2017
29	2	Secondary	Lombardy	Mantua	Fattening Turkeys	3-Aug-2017	9-Aug-2017	11-Aug-2017
30	2	Primary	Veneto	Verona	Fattening Turkeys	19-Aug-2017	21-Aug-2017	22-Aug-2017
31	2	Primary	Lombardy	Lodi	Game Birds (Pheasants)	10-Aug-2017	21-Aug-2017	24-Aug-2017
32	2	Primary	Veneto	Verona	Fattening Turkeys	20-Aug-2017	22-Aug-2017	23-Aug-2017
33	2	Primary	Veneto	Verona	Fattening Turkeys	20-Aug-2017	23-Aug-2017	24-Aug-2017
34	2	Primary	Lombardy	Pavia	Backyard	16-Aug-2017	25-Aug-2017	30-Aug-2017
35	2	Primary	Veneto	Verona	Fattening Turkeys	27-Aug-2017	28-Aug-2017	29-Aug-2017
36	2	Primary	Lombardy	Cremona	Fattening Turkeys	1-Sep-2017	2-Sep-2017	6-Sep-2017
37	2	Primary	Veneto	Padua	Geese	8-Sep-2017	11-Sep-2017	11-Sep-2017
38	2	Primary	Veneto	VIcenza	Fattening Turkeys	20-Sep-2017	26-Sep-2017	29-Sep-2017
39	2	Primary	Veneto	Verona	Fattening Turkeys	25-Sep-2017	26-Sep-2017	28-Sep-2017
40	2	Primary	Veneto	VIcenza	Ducks	24-Sep-2017	26-Sep-2017	28-Sep-2017
41	2	Primary	Lombardy	Cremona	Backyard	15-Sep-2017	28-Sep-2017	28-Sep-2017
42	2	Primary	Emilia Romagna	Ferrara	Laying Hens	4-Oct-2017	5-Oct-2017	18-Oct-2017
43	2	Secondary	Veneto	VIcenza	Chicken Broilers	2-Oct-2017	6-Oct-2017	9-Oct-2017
44	2	Secondary	Lombardy	Bergamo	Backyard	9-Oct-2017	10-Oct-2017	11-Oct-2017
45	2	Secondary	Veneto	VIcenza	Backyard	9-Oct-2017	10-Oct-2017	10-Oct-2017
46	2	Primary	Lombardy	Bergamo	Grower	11-Oct-2017	12-Oct-2017	12-Oct-2017
47	2	Primary	Veneto	Padua	Fattening Turkeys	11-Oct-2017	11-Oct-2017	13-Oct-2017
48	2	Primary	Lombardy	Brescia	Fattening Turkeys	9-Oct-2017	9-Oct-2017	11-Oct-2017
49	2	Secondary	Lombardy	Bergamo	Backyard	12-Oct-2017	13-Oct-2017	13-Oct-2017
50	2	Secondary	Lombardy	Brescia	Fattening Turkeys	13-Oct-2017	16-Oct-2017	16-Oct-2017
51	2	Secondary	Lombardy	Sondrio	Backyard	12-Oct-2017	16-Oct-2017	16-Oct-2017
52	2	Primary	Lombardy	Mantua	Fattening Turkeys	12-Oct-2017	13-Oct-2017	18-Oct-2017

ID	Wave	Type (Primary/Secondary)	Region	Province	Productive Type	Symptoms Onset	Confirmation Date	Extinction Date
53	2	Primary	Veneto	Padua	Backyard	13-Oct-2017	13-Oct-2017	16-Oct-2017
54	2	Primary	Veneto	Padua	Backyard	13-Oct-2017	13-Oct-2017	16-Oct-2017
55	2	Secondary	Lombardy	Brescia	Fattening Turkeys	18-Oct-2017	19-Oct-2017	24-Oct-2017
56	2	Secondary	Lombardy	Brescia	Fattening Turkeys	18-Oct-2017	19-Oct-2017	21-Oct-2017
57	2	Secondary	Lombardy	Bergamo	Backyard	20-Oct-2017	24-Oct-2017	24-Oct-2017
58	2	Primary	Lombardy	Milan	Backyard	24-Oct-2017	25-Oct-2017	25-Oct-2017
59	2	Secondary	Lombardy	Brescia	Ducks	25-Oct-2017	26-Oct-2017	28-Oct-2017
60	2	Secondary	Lombardy	Brescia	Chicken Broilers	26-Oct-2017	28-Oct-2017	2-Nov-2017
61	2	Secondary	Lombardy	Brescia	Chicken Broilers	27-Oct-2017	28-Oct-2017	3-Nov-2017
62	2	Secondary	Lombardy	Brescia	Fattening Turkeys	30-Oct-2017	31-Oct-2017	6-Nov-2017
63	2	Secondary	Lombardy	Brescia	Fattening Turkeys	31-Oct-2017	31-Oct-2017	7-Nov-2017
64	2	Secondary	Lombardy	Bergamo	Fattening Turkeys	31-Oct-2017	31-Oct-2017	5-Nov-2017
65	2	Secondary	Lombardy	Brescia	Fattening Turkeys	1-Nov-2017	2-Nov-2017	6-Nov-2017
66	2	Secondary	Lombardy	Brescia	Fattening Turkeys	1-Nov-2017	3-Nov-2017	7-Nov-2017
67	2	Secondary	Lombardy	Brescia	Ducks	26-Oct-2017	3-Nov-2017	8-Nov-2017
68	2	Secondary	Lombardy	Brescia	Ducks	1-Nov-2017	3-Nov-2017	8-Nov-2017
69	2	Secondary	Lombardy	Brescia	Fattening Turkeys	2-Nov-2017	6-Nov-2017	9-Nov-2017
70	2	Secondary	Lombardy	Brescia	Laying Hens	3-Nov-2017	6-Nov-2017	13-Nov-2017
71	2	Primary	Piedmont	Asti	Laying Hens	26-Oct-2017	6-Nov-2017	10-Nov-2017
72	2	Secondary	Lombardy	Brescia	Chicken Breeders	7-Nov-2017	8-Nov-2017	13-Nov-2017
73	2	Primary	Lazio	Roma	Backyard	2-Nov-2017	6-Nov-2017	28-Nov-2017
74	2	Secondary	Lombardy	Cremona	Fattening Turkeys	8-Nov-2017	8-Nov-2017	11-Nov-2017
75	2	Secondary	Lombardy	Brescia	Laying Hens	10-Nov-2017	10-Nov-2017	16-Nov-2017
76	2	Secondary	Lombardy	Brescia	Laying Hens	9-Nov-2017	9-Nov-2017	15-Nov-2017
77	2	Secondary	Lombardy	Brescia	Laying Hens	9-Nov-2017	10-Nov-2017	14-Nov-2017
78	2	Secondary	Lombardy	Brescia	Chicken Broilers	10-Nov-2017	10-Nov-2017	14-Nov-2017

ID	Wave	Type (Primary/Secondary)	Region	Province	Productive Type	Symptoms Onset	Confirmation Date	Extinction Date
79	2	Secondary	Lombardy	Brescia	Laying Hens	10-Nov-2017	10-Nov-2017	15-Nov-2017
80	2	Secondary	Lombardy	Brescia	Chicken Broilers	21-Nov-2017	22-Nov-2017	25-Nov-2017
81	2	Primary	Veneto	Treviso	Backyard	7-Nov-2017	23-Nov-2017	25-Nov-2017
82	2	Primary	Veneto	Treviso	Backyard	30-Nov-2017	1-Dec-2017	2-Dec-2017
83	2	Primary	Emilia Romagna	Ravenna	Fattening Turkeys	9-Dec-2017	11-Dec-2017	14-Dec-2017

Supplementary Table S2. Total number of nucleotide substitutions identified comparing the nucleotide sequences of each gene segment of the viruses collected within the same farm.

CASE	N. Sequences	PB2	PB1	PA	HA	NP	NA	M	NS
**6**	5	0	0	0	0	0	0	0	0
**18**	3	0	1	0	1	0	1	0	0
**19**	4	0	2	0	0	1	2	2	0
**21**	8	1	0	1	1	1	0	0	0
**24**	2	1	1	0	0	0	1	0	0
**26**	2	2	2	2	0	1	1	0	0
**29**	3	0	1	0	0	0	0	0	0
**30**	21	2	2	0	0	1	2	0	0
**37**	20	2	2	2	2	1	1	0	0
**40**	2	0	0	0	1	0	0	1	0
**41**	2	0	0	0	1	0	0	0	0
**42**	2	0	0	0	0	0	1	0	0
**43**	4	0	0	0	0	1	1	0	0
**45**	3	0	0	0	0	0	0	0	0
**48**	3	0	0	1	0	0	0	1	0
**50**	2	0	0	0	0	1	0	0	0
**57**	2	0	0	1	1	0	0	0	0
**82**	2	0	0	0	1	0	1	0	0

Supplementary Table S3. GISAID EpiFlu Database Acknowledgment table
We acknowledge the authors, originating and submitting laboratories of the sequences from GISAID's EpiFlu™ Database on which this research is based in part. The list is detailed below.
All submitters of data may be contacted directly via the GISAID website www.gisaid.org

Segment ID	Segment	Country	Isolate name	Originating Laboratory	Submitting Laboratory
EPI507673	HA	China	A/mallard duck/Shanghai/SH-9/2013	Institute of Military Veterinary, Academy of Military Medical Sciences	Institute of Laboratory Animal Sciences, Chinese Academy
EPI542617	HA	China	A/duck/Beijing/FS01/2013	Institute of Microbiology, Chinese Academy of Sciences	Institute of Microbiology, Chinese Academy of Sciences
EPI543002	HA	China	A/duck/Beijing/FS01/2014	Institute of Microbiology, Chinese Academy of Sciences	Institute of Microbiology, Chinese Academy of Sciences
EPI543010	HA	China	A/duck/Beijing/CT01/2014	Institute of Microbiology, Chinese Academy of Sciences	Institute of Microbiology, Chinese Academy of Sciences
EPI646105	HA	China	A/goose/Jiangsu/WX202/2014	Beijing Institute of Microbiology and Epidemiology	Beijing Institute of Microbiology and Epidemiology
EPI675774	HA	China	A/duck/Liaoning/S1001/2014	Harbin Veterinary Research Institute (CAAS)	Harbin Veterinary Research Institute (CAAS)
EPI774113	HA	China	A/Bar-headed Goose/Qinghai/BTY1-B/2016	State Key Laboratory of Virology and Wuhan Institute of Virology, Chinese Academy of Sciences	Wuhan Institute of Virology
EPI774121	HA	China	A/Bar-headed Goose/Qinghai/BTY1-LV/2016	State Key Laboratory of Virology and Wuhan Institute of Virology, Chinese Academy of Sciences	Wuhan Institute of Virology
EPI774133	HA	China	A/Bar-headed Goose/Qinghai/BTY2-B/2016	State Key Laboratory of Virology and Wuhan Institute of Virology, Chinese Academy of Sciences	Wuhan Institute of Virology
EPI774142	HA	China	A/Bar-headed Goose/Qinghai/BTY2-LU/2016	State Key Laboratory of Virology and Wuhan Institute of Virology, Chinese Academy of Sciences	Wuhan Institute of Virology
EPI774150	HA	China	A/Bar-headed Goose/Qinghai/BTY3-B/2016	State Key Laboratory of Virology and Wuhan Institute of Virology, Chinese Academy of Sciences	Wuhan Institute of Virology
EPI774159	HA	China	A/Bar-headed Goose/Qinghai/BTY3-LU/2016	State Key Laboratory of Virology and Wuhan Institute of Virology, Chinese Academy of Sciences	Wuhan Institute of Virology
EPI774168	HA	China	A/Bar-headed Goose/Qinghai/BTY4-B/2016	State Key Laboratory of Virology and Wuhan Institute of Virology, Chinese Academy of Sciences	Wuhan Institute of Virology
EPI774176	HA	China	A/Bar-headed Goose/Qinghai/BTY4-LU/2016	State Key Laboratory of Virology and Wuhan Institute of Virology, Chinese Academy of Sciences	Wuhan Institute of Virology

Segment ID	Segment	Country	Isolate name	Originating Laboratory	Submitting Laboratory
EPI774185	HA	China	A/Bar-headed Goose/Qinghai/BTY5-B/2016	State Key Laboratory of Virology and Wuhan Institute of Virology, Chinese Academy of Sciences	Wuhan Institute of Virology
EPI774193	HA	China	A/Bar-headed Goose/Qinghai/BTY6-B/2016	State Key Laboratory of Virology and Wuhan Institute of Virology, Chinese Academy of Sciences	Wuhan Institute of Virology
EPI774201	HA	China	A/Bar-headed Goose/Qinghai/BTY6-LU/2016	State Key Laboratory of Virology and Wuhan Institute of Virology, Chinese Academy of Sciences	Wuhan Institute of Virology
EPI774210	HA	China	A/Bar-headed Goose/Qinghai/BTY7-B/2016	State Key Laboratory of Virology and Wuhan Institute of Virology, Chinese Academy of Sciences	Wuhan Institute of Virology
EPI774218	HA	China	A/Bar-headed Goose/Qinghai/BTY7-LU1/2016	State Key Laboratory of Virology and Wuhan Institute of Virology, Chinese Academy of Sciences	Wuhan Institute of Virology
EPI774226	HA	China	A/Bar-headed Goose/Qinghai/BTY7-LU2/2016	State Key Laboratory of Virology and Wuhan Institute of Virology, Chinese Academy of Sciences	Wuhan Institute of Virology
EPI774234	HA	China	A/Bar-headed Goose/Qinghai/BTY8-B/2016	State Key Laboratory of Virology and Wuhan Institute of Virology, Chinese Academy of Sciences	Wuhan Institute of Virology
EPI774242	HA	China	A/Bar-headed Goose/Qinghai/BTY8-LU/2016	State Key Laboratory of Virology and Wuhan Institute of Virology, Chinese Academy of Sciences	Wuhan Institute of Virology
EPI774251	HA	China	A/Bar-headed Goose/Qinghai/BTY9-B/2016	State Key Laboratory of Virology and Wuhan Institute of Virology, Chinese Academy of Sciences	Wuhan Institute of Virology
EPI774259	HA	China	A/Bar-headed Goose/Qinghai/BTY9-LU/2016	State Key Laboratory of Virology and Wuhan Institute of Virology, Chinese Academy of Sciences	Wuhan Institute of Virology
EPI774267	HA	China	A/Bar-headed Goose/Qinghai/BTY10-B/2016	State Key Laboratory of Virology and Wuhan Institute of Virology, Chinese Academy of Sciences	Wuhan Institute of Virology
EPI774277	HA	China	A/Bar-headed Goose/Qinghai/BTY10-LU/2016	State Key Laboratory of Virology and Wuhan Institute of Virology, Chinese Academy of Sciences	Wuhan Institute of Virology
EPI774286	HA	China	A/Bar-headed Goose/Qinghai/BTY11-B/2016	State Key Laboratory of Virology and Wuhan Institute of Virology, Chinese Academy of Sciences	Wuhan Institute of Virology
EPI774294	HA	China	A/Bar-headed Goose/Qinghai/BTY11-LU/2016	State Key Laboratory of Virology and Wuhan Institute of Virology, Chinese Academy of Sciences	Wuhan Institute of Virology
EPI774302	HA	China	A/Bar-headed Goose/Qinghai/BTY12-B/2016	State Key Laboratory of Virology and Wuhan Institute of Virology, Chinese Academy of Sciences	Wuhan Institute of Virology

Segment ID	Segment	Country	Isolate name	Originating Laboratory	Submitting Laboratory
EPI774310	HA	China	A/Bar-headed Goose/Qinghai/BTY12-LU/2016	State Key Laboratory of Virology and Wuhan Institute of Virology, Chinese Academy of Sciences	Wuhan Institute of Virology
EPI774318	HA	China	A/Bar-headed Goose/Qinghai/BTY13-B/2016	State Key Laboratory of Virology and Wuhan Institute of Virology, Chinese Academy of Sciences	Wuhan Institute of Virology
EPI774326	HA	China	A/Bar-headed Goose/Qinghai/BTY13-LU/2016	State Key Laboratory of Virology and Wuhan Institute of Virology, Chinese Academy of Sciences	Wuhan Institute of Virology
EPI774335	HA	China	A/Bar-headed Goose/Qinghai/BTY14-B/2016	State Key Laboratory of Virology and Wuhan Institute of Virology, Chinese Academy of Sciences	Wuhan Institute of Virology
EPI774344	HA	China	A/Bar-headed Goose/Qinghai/BTY14-LU/2016	State Key Laboratory of Virology and Wuhan Institute of Virology, Chinese Academy of Sciences	Wuhan Institute of Virology
EPI774352	HA	China	A/Bar-headed Goose/Qinghai/BTY15-B/2016	State Key Laboratory of Virology and Wuhan Institute of Virology, Chinese Academy of Sciences	Wuhan Institute of Virology
EPI774361	HA	China	A/Bar-headed Goose/Qinghai/BTY15-LU/2016	State Key Laboratory of Virology and Wuhan Institute of Virology, Chinese Academy of Sciences	Wuhan Institute of Virology
EPI774369	HA	China	A/Bar-headed Goose/Qinghai/BTY16-B/2016	State Key Laboratory of Virology and Wuhan Institute of Virology, Chinese Academy of Sciences	Wuhan Institute of Virology
EPI774378	HA	China	A/Bar-headed Goose/Qinghai/BTY16-LU/2016	State Key Laboratory of Virology and Wuhan Institute of Virology, Chinese Academy of Sciences	Wuhan Institute of Virology
EPI774386	HA	China	A/Bar-headed Goose/Qinghai/BTY17-B/2016	State Key Laboratory of Virology and Wuhan Institute of Virology, Chinese Academy of Sciences	Wuhan Institute of Virology
EPI774394	HA	China	A/Bar-headed Goose/Qinghai/BTY17-LU/2016	State Key Laboratory of Virology and Wuhan Institute of Virology, Chinese Academy of Sciences	Wuhan Institute of Virology
EPI774402	HA	China	A/Bar-headed Goose/Qinghai/BTY18-B/2016	State Key Laboratory of Virology and Wuhan Institute of Virology, Chinese Academy of Sciences	Wuhan Institute of Virology
EPI774410	HA	China	A/Bar-headed Goose/Qinghai/BTY18-LU/2016	State Key Laboratory of Virology and Wuhan Institute of Virology, Chinese Academy of Sciences	Wuhan Institute of Virology
EPI774426	HA	China	A/Brown-headed Gull/Qinghai/ZTO1-B/2016	State Key Laboratory of Virology and Wuhan Institute of Virology, Chinese Academy of Sciences	Wuhan Institute of Virology
EPI774434	HA	China	A/Brown-headed Gull/Qinghai/ZTO1-LU/2016	State Key Laboratory of Virology and Wuhan Institute of Virology, Chinese Academy of Sciences	Wuhan Institute of Virology

Segment ID	Segment	Country	Isolate name	Originating Laboratory	Submitting Laboratory
EPI774442	HA	China	A/Brown-headed Gull/Qinghai/ZTO3-B/2016	State Key Laboratory of Virology and Wuhan Institute of Virology, Chinese Academy of Sciences	Wuhan Institute of Virology
EPI774450	HA	China	A/Brown-headed Gull/Qinghai/ZTO3-LU/2016	State Key Laboratory of Virology and Wuhan Institute of Virology, Chinese Academy of Sciences	Wuhan Institute of Virology
EPI774458	HA	China	A/Brown-headed Gull/Qinghai/ZTO4-B/2016	State Key Laboratory of Virology and Wuhan Institute of Virology, Chinese Academy of Sciences	Wuhan Institute of Virology
EPI774466	HA	China	A/Brown-headed Gull/Qinghai/ZTO5-B/2016	State Key Laboratory of Virology and Wuhan Institute of Virology, Chinese Academy of Sciences	Wuhan Institute of Virology
EPI774475	HA	China	A/Brown-headed Gull/Qinghai/ZTO5-K/2016	State Key Laboratory of Virology and Wuhan Institute of Virology, Chinese Academy of Sciences	Wuhan Institute of Virology
EPI774483	HA	China	A/Brown-headed Gull/Qinghai/ZTO6-B/2016	State Key Laboratory of Virology and Wuhan Institute of Virology, Chinese Academy of Sciences	Wuhan Institute of Virology
EPI774490	HA	China	A/Brown-headed Gull/Qinghai/ZTO6-SP/2016	State Key Laboratory of Virology and Wuhan Institute of Virology, Chinese Academy of Sciences	Wuhan Institute of Virology
EPI774498	HA	China	A/Brown-headed Gull/Qinghai/ZTO6-MU/2016	State Key Laboratory of Virology and Wuhan Institute of Virology, Chinese Academy of Sciences	Wuhan Institute of Virology
EPI774506	HA	China	A/Great Black-headed Gull/Qinghai/YO1-B/2016	State Key Laboratory of Virology and Wuhan Institute of Virology, Chinese Academy of Sciences	Wuhan Institute of Virology
EPI861568	HA	Croatia	A/mute swan/Croatia/70/2016	NA	Croatian Veterinary Institute
EPI861572	HA	Croatia	A/mute swan/Croatia/78/2016	NA	Croatian Veterinary Institute
EPI864746	HA	Croatia	A/mute swan/Croatia/85/2016	NA	Croatian Veterinary Institute
EPI873212	HA	Croatia	A/chicken/Croatia/103/2016	NA	Croatian Veterinary Institute
EPI873624	HA	Croatia	A/mute swan/Croatia/9/2017	NA	Croatian Veterinary Institute
EPI873626	HA	Croatia	A/mute swan/Croatia/104/2016	NA	Croatian Veterinary Institute

Segment ID	Segment	Country	Isolate name	Originating Laboratory	Submitting Laboratory
EPI881899	HA	Croatia	A/breeder duck/Croatia/21/2017	NA	Croatian Veterinary Institute
EPI881901	HA	Croatia	A/mute swan/Croatia/15/2017	NA	Croatian Veterinary Institute
EPI891667	HA	Croatia	A/mute swan/Croatia/30/2017	NA	Croatian Veterinary Institute
EPI891670	HA	Croatia	A/greylag goose/Croatia/33/2017	NA	Croatian Veterinary Institute
EPI917275	HA	Croatia	A/mute swan/Croatia/61/2017	NA	Croatian Veterinary Institute
EPI917277	HA	Croatia	A/chicken/Croatia/70/2017	NA	Croatian Veterinary Institute
EPI1021086	HA	Czech Republic	A/mute swan/Czech Republic/54-17_1/2017 (H5N8)	State Veterinary Institute Prague	State Veterinary Institute Prague
EPI1021087	HA	Czech Republic	A/goose/Czech Republic/136-17_1/2017 (H5N8)	State Veterinary Institute Prague	State Veterinary Institute Prague
EPI1021088	HA	Czech Republic	A/mallard/Czech Republic/136-17_2/2017 (H5N8)	State Veterinary Institute Prague	State Veterinary Institute Prague
EPI1021089	HA	Czech Republic	A/goose/Czech Republic/197-17/2017 (H5N8)	State Veterinary Institute Prague	State Veterinary Institute Prague
EPI1021090	HA	Czech Republic	A/chicken/Czech Republic/508-17_1/2017 (H5N8)	State Veterinary Institute Prague	State Veterinary Institute Prague
EPI1021091	HA	Czech Republic	A/mallard/Czech Republic/508-17_4/2017 (H5N8)	State Veterinary Institute Prague	State Veterinary Institute Prague
EPI1021092	HA	Czech Republic	A/mute swan/Czech Republic/572-17_3/2017 (H5N8)	State Veterinary Institute Prague	State Veterinary Institute Prague
EPI1021093	HA	Czech Republic	A/mute swan/Czech Republic/964-17/2017 (H5N8)	State Veterinary Institute Prague	State Veterinary Institute Prague
EPI1021094	HA	Czech Republic	A/mute swan/Czech Republic/967-17/2017 (H5N8)	State Veterinary Institute Prague	State Veterinary Institute Prague

Segment ID	Segment	Country	Isolate name	Originating Laboratory	Submitting Laboratory
EPI1021095	HA	Czech Republic	A/mute swan/Czech Republic/987-17_2/2017 (H5N8)	State Veterinary Institute Prague	State Veterinary Institute Prague
EPI1021096	HA	Czech Republic	A/mute swan/Czech Republic/1058-17/2017 (H5N8)	State Veterinary Institute Prague	State Veterinary Institute Prague
EPI1021097	HA	Czech Republic	A/mute swan/Czech Republic/1060-17/2017 (H5N8)	State Veterinary Institute Prague	State Veterinary Institute Prague
EPI1021098	HA	Czech Republic	A/mute swan/Czech Republic/1155-17/2017 (H5N8)	State Veterinary Institute Prague	State Veterinary Institute Prague
EPI1021099	HA	Czech Republic	A/mute swan/Czech Republic/1156-17/2017 (H5N8)	State Veterinary Institute Prague	State Veterinary Institute Prague
EPI1021100	HA	Czech Republic	A/mute swan/Czech Republic/1170-17_2/2017 (H5N8)	State Veterinary Institute Prague	State Veterinary Institute Prague
EPI1021101	HA	Czech Republic	A/mute swan/Czech Republic/1171-17/2017 (H5N8)	State Veterinary Institute Prague	State Veterinary Institute Prague
EPI1021102	HA	Czech Republic	A/chicken/Czech Republic/1208-17_1/2017 (H5N8)	State Veterinary Institute Prague	State Veterinary Institute Prague
EPI1021103	HA	Czech Republic	A/mallard/Czech Republic/1219-17_1/2017 (H5N8)	State Veterinary Institute Prague	State Veterinary Institute Prague
EPI1021104	HA	Czech Republic	A/mallard/Czech Republic/1226-17/2017 (H5N8)	State Veterinary Institute Prague	State Veterinary Institute Prague
EPI1021105	HA	Czech Republic	A/mute swan/Czech Republic/1227-17/2017 (H5N8)	State Veterinary Institute Prague	State Veterinary Institute Prague
EPI1021106	HA	Czech Republic	A/mute swan/Czech Republic/1296-17_1/2017 (H5N8)	State Veterinary Institute Prague	State Veterinary Institute Prague
EPI1021107	HA	Czech Republic	A/mute swan/Czech Republic/1330-17_1/2017 (H5N8)	State Veterinary Institute Prague	State Veterinary Institute Prague
EPI1021108	HA	Czech Republic	A/mute swan/Czech Republic/1331-17_1/2017 (H5N8)	State Veterinary Institute Prague	State Veterinary Institute Prague
EPI1021109	HA	Czech Republic	A/mute swan/Czech Republic/1337-17/2017 (H5N8)	State Veterinary Institute Prague	State Veterinary Institute Prague

Segment ID	Segment	Country	Isolate name	Originating Laboratory	Submitting Laboratory
EPI1021110	HA	Czech Republic	A/mute swan/Czech Republic/1339-17/2017 (H5N8)	State Veterinary Institute Prague	State Veterinary Institute Prague
EPI1021111	HA	Czech Republic	A/chicken/Czech Republic/1344-17/2017 (H5N8)	State Veterinary Institute Prague	State Veterinary Institute Prague
EPI1021112	HA	Czech Republic	A/mute swan/Czech Republic/1461-17/2017 (H5N8)	State Veterinary Institute Prague	State Veterinary Institute Prague
EPI1021113	HA	Czech Republic	A/chicken/Czech Republic/1465-17/2017 (H5N8)	State Veterinary Institute Prague	State Veterinary Institute Prague
EPI1021114	HA	Czech Republic	A/mute swan/Czech Republic/1519-17/2017 (H5N8)	State Veterinary Institute Prague	State Veterinary Institute Prague
EPI1021115	HA	Czech Republic	A/mute swan/Czech Republic/1576-17_C/2017 (H5N8)	State Veterinary Institute Prague	State Veterinary Institute Prague
EPI1021116	HA	Czech Republic	A/mallard/Czech Republic/1577-17/2017 (H5N8)	State Veterinary Institute Prague	State Veterinary Institute Prague
EPI1021117	HA	Czech Republic	A/mute swan/Czech Republic/1640-17/2017 (H5N8)	State Veterinary Institute Prague	State Veterinary Institute Prague
EPI1021118	HA	Czech Republic	A/mallard/Czech Republic/1672-17/2017 (H5N8)	State Veterinary Institute Prague	State Veterinary Institute Prague
EPI1021119	HA	Czech Republic	A/chicken/Czech Republic/1675-17_2/2017 (H5N8)	State Veterinary Institute Prague	State Veterinary Institute Prague
EPI1021120	HA	Czech Republic	A/grey heron/Czech Republic/1680-17_2/2017 (H5N8)	State Veterinary Institute Prague	State Veterinary Institute Prague
EPI1021121	HA	Czech Republic	A/Indian Runner Duck/Czech Republic/1683-17_1/2017 (H5N8)	State Veterinary Institute Prague	State Veterinary Institute Prague
EPI1021122	HA	Czech Republic	A/chicken/Czech Republic/1687-17_2/2017 (H5N8)	State Veterinary Institute Prague	State Veterinary Institute Prague
EPI1021123	HA	Czech Republic	A/chicken/Czech Republic/1688-17_1/2017 (H5N8)	State Veterinary Institute Prague	State Veterinary Institute Prague
EPI1021124	HA	Czech Republic	A/chicken/Czech Republic/1689-17/2017 (H5N8)	State Veterinary Institute Prague	State Veterinary Institute Prague

Segment ID	Segment	Country	Isolate name	Originating Laboratory	Submitting Laboratory
EPI1021125	HA	Czech Republic	A/mallard/Czech Republic/1690-17_2/2017 (H5N8)	State Veterinary Institute Prague	State Veterinary Institute Prague
EPI1021126	HA	Czech Republic	A/mute swan/Czech Republic/1691-17/2017 (H5N8)	State Veterinary Institute Prague	State Veterinary Institute Prague
EPI1021127	HA	Czech Republic	A/bronze turkey/Czech Republic/1755-17_1/2017 (H5N8)	State Veterinary Institute Prague	State Veterinary Institute Prague
EPI1021128	HA	Czech Republic	A/turkey/Czech Republic/1767-17_2/2017 (H5N8)	State Veterinary Institute Prague	State Veterinary Institute Prague
EPI1021129	HA	Czech Republic	A/mute swan/Czech Republic/1848-17_1/2017 (H5N8)	State Veterinary Institute Prague	State Veterinary Institute Prague
EPI1021130	HA	Czech Republic	A/mute swan/Czech Republic/1848-17_2/2017 (H5N8)	State Veterinary Institute Prague	State Veterinary Institute Prague
EPI1021131	HA	Czech Republic	A/chicken/Czech Republic/1953-17/2017 (H5N8)	State Veterinary Institute Prague	State Veterinary Institute Prague
EPI1021132	HA	Czech Republic	A/goose/Czech Republic/1954-17/2017 (H5N8)	State Veterinary Institute Prague	State Veterinary Institute Prague
EPI1021133	HA	Czech Republic	A/goose/Czech Republic/1998-17_1/2017 (H5N8)	State Veterinary Institute Prague	State Veterinary Institute Prague
EPI1021134	HA	Czech Republic	A/mute swan/Czech Republic/2008-17_1/2017 (H5N8)	State Veterinary Institute Prague	State Veterinary Institute Prague
EPI1021135	HA	Czech Republic	A/quail/Czech Republic/2063-17_1/2017 (H5N8)	State Veterinary Institute Prague	State Veterinary Institute Prague
EPI1021136	HA	Czech Republic	A/chicken/Czech Republic/2216-17_1/2017 (H5N8)	State Veterinary Institute Prague	State Veterinary Institute Prague
EPI1021138	HA	Czech Republic	A/chicken/Czech Republic/2514-17/2017 (H5N8)	State Veterinary Institute Prague	State Veterinary Institute Prague
EPI1021139	HA	Czech Republic	A/mallard/Czech Republic/2641-17/2017 (H5N8)	State Veterinary Institute Prague	State Veterinary Institute Prague
EPI1021140	HA	Czech Republic	A/chicken/Czech Republic/2677-17_2/2017 (H5N8)	State Veterinary Institute Prague	State Veterinary Institute Prague

Segment ID	Segment	Country	Isolate name	Originating Laboratory	Submitting Laboratory
EPI1021141	HA	Czech Republic	A/mallard/Czech Republic/2678-17_1/2017 (H5N8)	State Veterinary Institute Prague	State Veterinary Institute Prague
EPI1021142	HA	Czech Republic	A/mallard/Czech Republic/2820-17_1/2017 (H5N8)	State Veterinary Institute Prague	State Veterinary Institute Prague
EPI1021143	HA	Czech Republic	A/mallard/Czech Republic/2820-17_2/2017 (H5N8)	State Veterinary Institute Prague	State Veterinary Institute Prague
EPI1021144	HA	Czech Republic	A/mallard/Czech Republic/2821-17_1/2017 (H5N8)	State Veterinary Institute Prague	State Veterinary Institute Prague
EPI1021145	HA	Czech Republic	A/chicken/Czech Republic/2821-17_2/2017 (H5N8)	State Veterinary Institute Prague	State Veterinary Institute Prague
EPI1021146	HA	Czech Republic	A/mallard/Czech Republic/2822-17_1/2017 (H5N8)	State Veterinary Institute Prague	State Veterinary Institute Prague
EPI1021147	HA	Czech Republic	A/chicken/Czech Republic/2822-17_2/2017 (H5N8)	State Veterinary Institute Prague	State Veterinary Institute Prague
EPI1021148	HA	Czech Republic	A/chicken/Czech Republic/3507-17/2017 (H5N8)	State Veterinary Institute Prague	State Veterinary Institute Prague
EPI1081894	HA	Czech Republic	A/turkey/Czech Republic/38-17_1/2017 (H5N8)	State Veterinary Institute Prague	State Veterinary Institute Prague
EPI1087636	HA	Czech Republic	A/chicken/Czech Republic/988-17/2017 (H5N8)	State Veterinary Institute Prague	State Veterinary Institute Prague
EPI887468	HA	Czech Republic	A/chicken/Czech Republic/206-17_2/2017(H5N8)	NA	State Veterinary Institute Prague
EPI888277	HA	Czech Republic	A/turkey/Czech Republic/38-17_5/2017 (H5N8)	NA	State Veterinary Institute Prague
EPI916385	HA	Czech Republic	A/mute swan/Czech Republic/653-17/2017 (H5N8)	State Veterinary Institute Prague	State Veterinary Institute Prague
EPI916503	HA	Czech Republic	A/mallard/Czech Republic/722-17_2/2017 (H5N8)	NA	State Veterinary Institute Prague
EPI916604	HA	Czech Republic	A/bronze turkey/Czech Republic/1414-17/2017 (H5N8)	NA	State Veterinary Institute Prague

Segment ID	Segment	Country	Isolate name	Originating Laboratory	Submitting Laboratory
EPI917480	HA	Czech Republic	A/mute swan/Czech Republic/722-17_1/2017 (H5N8)	State Veterinary Institute Prague	State Veterinary Institute Prague
EPI919636	HA	Czech Republic	A/chicken/Czech Republic/585-17_1/2017 (H5N8)	State Veterinary Institute Prague	State Veterinary Institute Prague
EPI930838	HA	Czech Republic	A/mute swan/Czech Republic/54-17_2/2017 (H5N8)	NA	State Veterinary Institute Prague
EPI930841	HA	Czech Republic	A/Indian Runner Duck/Czech Republic/749-17/2017 (H5N8)	NA	State Veterinary Institute Prague
EPI930843	HA	Czech Republic	A/chicken/Czech Republic/55-17_1/2017 (H5N8)	NA	State Veterinary Institute Prague
EPI931110	HA	Czech Republic	A/mute swan/Czech Republic/879-17/2017 (H5N8)	NA	State Veterinary Institute Prague
EPI931145	HA	Czech Republic	A/duck/Czech Republic/1467-17/2017 (H5N8)	NA	State Veterinary Institute Prague
EPI931194	HA	Czech Republic	A/chicken/Czech Republic/1863-17_1/2017 (H5N8)	NA	State Veterinary Institute Prague
EPI931197	HA	Czech Republic	A/chicken/Czech Republic/1896-17_1/2017 (H5N8)	NA	State Veterinary Institute Prague
EPI942419	HA	Czech Republic	A/chicken/Czech Republic/2764-17_1/2017 (H5N8)	NA	State Veterinary Institute Prague
EPI942420	HA	Czech Republic	A/chicken/Czech Republic/2764-17_2/2017 (H5N8)	NA	State Veterinary Institute Prague
EPI961475	HA	Czech Republic	A/mute swan/Czech Republic/499-17/2017 (H5N8)	NA	State Veterinary Institute Prague
EPI961484	HA	Czech Republic	A/mute swan/Czech Republic/581-17/2017 (H5N8)	NA	State Veterinary Institute Prague
EPI961528	HA	Czech Republic	A/goose/Czech Republic/821-17_2/2017 (H5N8)	NA	State Veterinary Institute Prague
EPI969259	HA	Czech Republic	A/mute swan/Czech Republic/1813-17/2017 (H5N8)	NA	State Veterinary Institute Prague

Segment ID	Segment	Country	Isolate name	Originating Laboratory	Submitting Laboratory
EPI969262	HA	Czech Republic	A/chicken/Czech Republic/2643-17_1/2017 (H5N8)	NA	State Veterinary Institute Prague
EPI969265	HA	Czech Republic	A/chicken/Czech Republic/2644-17_1/2017 (H5N8)	NA	State Veterinary Institute Prague
EPI969268	HA	Czech Republic	A/mallard/Czech Republic/2705-17/2017 (H5N8)	NA	State Veterinary Institute Prague
EPI860239	HA	Denmark	A/tufted duck/Denmark/17740-1/2016	Technical University of Denmark	Technical University of Denmark
EPI909364	HA	Denmark	A/tufted duck/Denmark/17740-1/2016	Technical University of Denmark	Animal and Plant Health Agency (APHA)
EPI868854	HA	Egypt	A/Common-coot/Egypt/CA285/2016	National Laboratory for Veterinary Quality Control on Poultry production- Animal Health Research Inititute	Animal Health Research Institute
EPI869687	HA	France	A/decoy_duck/France/161104e/2016	Anses (Ploufragan-Plouzané)	French Agency for Food, Environmental and Occupational Health
EPI869809	HA	France	A/duck/France/161108h/2016		French Agency for Food, Environmental and Occupational Health
EPI544756	HA	Germany	A/turkey/Germany-MV/R2472/2014	NA	Friedrich-Loeffler-Institut
EPI552746	HA	Germany	A/turkey/Germany/AR2485-86-L00899/2014	NA	Friedrich-Loeffler-Institut
EPI553145	HA	Germany	A/turkey/Germany-NI/R3372/2014	NA	Friedrich-Loeffler-Institut
EPI553172	HA	Germany	A/turkey/Germany-NI/R3372/2014	NA	Friedrich-Loeffler-Institut
EPI554605	HA	Germany	A/domestic duck/Germany-NI/R3468/2014	NA	Friedrich-Loeffler-Institut
EPI554607	HA	Germany	A/mallard/Germany-ST/R23/2015	NA	Friedrich-Loeffler-Institut
EPI555126	HA	Germany	A/stork/Germany-MV/R24/2015	NA	Friedrich-Loeffler-Institut

Segment ID	Segment	Country	Isolate name	Originating Laboratory	Submitting Laboratory
EPI556967	HA	Germany	A/chicken/Germany-MV/R153/2015	NA	Friedrich-Loeffler-Institut
EPI566027	HA	Germany	A/ibis/Germany-MV/R44/2015	NA	Friedrich-Loeffler-Institut
EPI624535	HA	Germany	A/scarlet_ibis/Germany/AR44-L01279/2015	NA	Friedrich-Loeffler-Institut
EPI687239	HA	Germany	A/turkey/Germany/AR3390-L00939/2014	NA	Friedrich-Loeffler-Institut
EPI687247	HA	Germany	A/turkey/Germany/AR3382-L00937/2014	NA	Friedrich-Loeffler-Institut
EPI691836	HA	Germany	A/gull/Germany-NI/R45/2015	NA	Friedrich-Loeffler-Institut
EPI859212	HA	Germany	A/tufted_duck/Germany-SH/R8446/2016	NA	Friedrich-Loeffler-Institut
EPI859650	HA	Germany	A/wild duck/Germany-BW/R8455/2016	NA	Friedrich-Loeffler-Institut
EPI859653	HA	Germany	A/tufted_duck/Germany/AR8444-L01986/2016	NA	Friedrich-Loeffler-Institut
EPI860393	HA	Germany	A/tufted_duck/Germany/AR8444-L01987/2016	NA	Friedrich-Loeffler-Institut
EPI860401	HA	Germany	A/tufted_duck/Germany/AR8459-L01988/2016	NA	Friedrich-Loeffler-Institut
EPI860509	HA	Germany	A/tufted duck/Germany-SH/R8444/2016	NA	Friedrich-Loeffler-Institut
EPI861011	HA	Germany	A/turkey/Germany-SH/R8595/2016	NA	Friedrich-Loeffler-Institut
EPI861012	HA	Germany	A/chicken/Germany-MV/R8790/2016	NA	Friedrich-Loeffler-Institut
EPI861222	HA	Germany	A/tufted duck/Germany-SN/R8795/2016	NA	Friedrich-Loeffler-Institut

Segment ID	Segment	Country	Isolate name	Originating Laboratory	Submitting Laboratory
EPI861224	HA	Germany	A/chicken/Germany-SH/R8758/2016	NA	Friedrich-Loeffler-Institut
EPI876474	HA	Germany	A/turkey/Germany-NI/R10523/2016	NA	Friedrich-Loeffler-Institut
EPI881292	HA	Germany	A/domestic duck/Germany-MV/R9869/2016	NA	Friedrich-Loeffler-Institut
EPI907346	HA	Germany	A/domestic duck/Germany-BB/R681ff/2017	NA	Friedrich-Loeffler-Institut
EPI922444	HA	Germany	A/domestic goose/Germany-BY/R677/2017	NA	Friedrich-Loeffler-Institut
EPI922508	HA	Germany	A/chicken/Germany-NI/R11406/2016	NA	Friedrich-Loeffler-Institut
EPI931207	HA	Germany	A/turkey/Germany-BB/R234ff/2017	NA	Friedrich-Loeffler-Institut
EPI932938	HA	Germany	A/chicken/Germany-MV/R10048/2016	NA	Friedrich-Loeffler-Institut
EPI963516	HA	Germany	A/domestic duck/Germany-MV/R9764/2016	NA	Friedrich-Loeffler-Institut
EPI967465	HA	Germany	A/turkey/Germany-NI/R9807/2016	NA	Friedrich-Loeffler-Institut
EPI969347	HA	Germany	A/turkey/Germany-BB/R377ff/2017	NA	Friedrich-Loeffler-Institut
EPI973841	HA	Germany	A/white stork/Germany-TH/R1149/2017	NA	Friedrich-Loeffler-Institut
EPI973844	HA	Germany	A/tawny owl/Germany-SN/R1186/2017	NA	Friedrich-Loeffler-Institut
EPI978863	HA	Germany	A/grey heron/Germany-TH/R1125/2017	NA	Friedrich-Loeffler-Institut
EPI978865	HA	Germany	A/mute swan/Germany-TH/R1126/2017	NA	Friedrich-Loeffler-Institut

Segment ID	Segment	Country	Isolate name	Originating Laboratory	Submitting Laboratory
EPI988345	HA	Germany	A/black swan/Germany-BW/R1364/2017	NA	Friedrich-Loeffler-Institut
EPI990770	HA	Germany	A/eurasian wigeon/Germany-NI/AR249-L02143/2017	NA	Friedrich-Loeffler-Institut
EPI990786	HA	Germany	A/mute swan/Germany-NI/AR1529-L02145/2017	NA	Friedrich-Loeffler-Institut
EPI990794	HA	Germany	A/greylag goose/Germany-NI/AR1395-L02144/2017	NA	Friedrich-Loeffler-Institut
EPI990802	HA	Germany	A/greylag goose/Germany-NI/AR703-L02138/2017	NA	Friedrich-Loeffler-Institut
EPI1032476	HA	Hungary	A/Goose/Hungary/17261/2017	National Food Chain Safety Office Veterinary Diagnostic Directorate Laboratory for Molecular Biology	Central Agricultural Office Veterinary Diagnostic Directorate
EPI1032484	HA	Hungary	A/Goose/Hungary/15729/2017	National Food Chain Safety Office Veterinary Diagnostic Directorate Laboratory for Molecular Biology	Central Agricultural Office Veterinary Diagnostic Directorate
EPI1032492	HA	Hungary	A/Goose/Hungary/17051/2017	National Food Chain Safety Office Veterinary Diagnostic Directorate Laboratory for Molecular Biology	Central Agricultural Office Veterinary Diagnostic Directorate
EPI1032500	HA	Hungary	A/Goose/Hungary/17580/2017	National Food Chain Safety Office Veterinary Diagnostic Directorate Laboratory for Molecular Biology	Central Agricultural Office Veterinary Diagnostic Directorate
EPI1032508	HA	Hungary	A/Goose/Hungary/17985/2017	National Food Chain Safety Office Veterinary Diagnostic Directorate Laboratory for Molecular Biology	Central Agricultural Office Veterinary Diagnostic Directorate
EPI1032516	HA	Hungary	A/Goose/Hungary/59763/2016	National Food Chain Safety Office Veterinary Diagnostic Directorate Laboratory for Molecular Biology	Central Agricultural Office Veterinary Diagnostic Directorate
EPI1032524	HA	Hungary	A/Mulard_duck/Hungary/60369/2016	National Food Chain Safety Office Veterinary Diagnostic Directorate Laboratory for Molecular Biology	Central Agricultural Office Veterinary Diagnostic Directorate
EPI1032532	HA	Hungary	A/Mulard_duck/Hungary/62902/2016	National Food Chain Safety Office Veterinary Diagnostic Directorate Laboratory for Molecular Biology	Central Agricultural Office Veterinary Diagnostic Directorate
EPI1032540	HA	Hungary	A/Goose/Hungary/63743/2016	National Food Chain Safety Office Veterinary Diagnostic Directorate Laboratory for Molecular Biology	Central Agricultural Office Veterinary Diagnostic Directorate
EPI1032548	HA	Hungary	A/Goose/Hungary/64909/2016	National Food Chain Safety Office Veterinary Diagnostic Directorate Laboratory for Molecular Biology	Central Agricultural Office Veterinary Diagnostic Directorate

Segment ID	Segment	Country	Isolate name	Originating Laboratory	Submitting Laboratory
EPI1032549	HA	Hungary	A/Mulard_duck/Hungary/59163/2016	National Food Chain Safety Office Veterinary Diagnostic Directorate Laboratory for Molecular Biology	Central Agricultural Office Veterinary Diagnostic Directorate
EPI584823	HA	Hungary	A/domestic duck/Hungary/7341/2015	Central Agricultural Office Veterinary Diagnostic Directorate	Central Agricultural Office Veterinary Diagnostic Directorate
EPI859199	HA	Hungary	A/mute_swan/Hungary/51049/2016	National Food Chain Safety Office Veterinary Diagnostic Directorate Laboratory for Molecular Biology	Central Agricultural Office Veterinary Diagnostic Directorate
EPI859207	HA	Hungary	A/domestic_turkey/Hungary/53433/2016	National Food Chain Safety Office Veterinary Diagnostic Directorate Laboratory for Molecular Biology	Central Agricultural Office Veterinary Diagnostic Directorate
EPI860518	HA	Hungary	A/Mulard_duck/Hungary/54494/2016	National Food Chain Safety Office Veterinary Diagnostic Directorate Laboratory for Molecular Biology	Central Agricultural Office Veterinary Diagnostic Directorate
EPI860519	HA	Hungary	A/goose/Hungary/55128/2016	National Food Chain Safety Office Veterinary Diagnostic Directorate Laboratory for Molecular Biology	Central Agricultural Office Veterinary Diagnostic Directorate
EPI860534	HA	Hungary	A/duck/Hungary/55191/2016	National Food Chain Safety Office Veterinary Diagnostic Directorate Laboratory for Molecular Biology	Central Agricultural Office Veterinary Diagnostic Directorate
EPI866976	HA	Hungary	A/chicken/Hungary/59048/2016	National Food Chain Safety Office Veterinary Diagnostic Directorate Laboratory for Molecular Biology	Central Agricultural Office Veterinary Diagnostic Directorate
EPI866978	HA	Hungary	A/duck/Hungary/60441/2016	National Food Chain Safety Office Veterinary Diagnostic Directorate Laboratory for Molecular Biology	Central Agricultural Office Veterinary Diagnostic Directorate
EPI953337	HA	Hungary	A/mallard/Hungary/57857/2016	Central Agricultural Office Veterinary Diagnostic Directorate	Central Agricultural Office Veterinary Diagnostic Directorate
EPI954539	HA	Hungary	A/Goose/Hungary/65817/2016	Central Agricultural Office Veterinary Diagnostic Directorate	Central Agricultural Office Veterinary Diagnostic Directorate
EPI954631	HA	Hungary	A/Mute swan/Hungary/119/2017	Central Agricultural Office Veterinary Diagnostic Directorate	Central Agricultural Office Veterinary Diagnostic Directorate
EPI954639	HA	Hungary	A/Harris Hawk/Hungary/120/2017	Central Agricultural Office Veterinary Diagnostic Directorate	Central Agricultural Office Veterinary Diagnostic Directorate
EPI954647	HA	Hungary	A/Goose/Hungary/982/2017	Central Agricultural Office Veterinary Diagnostic Directorate	Central Agricultural Office Veterinary Diagnostic Directorate
EPI954655	HA	Hungary	A/Duck/Hungary/984/2017	Central Agricultural Office Veterinary Diagnostic Directorate	Central Agricultural Office Veterinary Diagnostic Directorate

Segment ID	Segment	Country	Isolate name	Originating Laboratory	Submitting Laboratory
EPI954663	HA	Hungary	A/Goose/Hungary/1030/2017	Central Agricultural Office Veterinary Diagnostic Directorate	Central Agricultural Office Veterinary Diagnostic Directorate
EPI954671	HA	Hungary	A/Duck/Hungary/1588/2017	Central Agricultural Office Veterinary Diagnostic Directorate	Central Agricultural Office Veterinary Diagnostic Directorate
EPI954679	HA	Hungary	A/Greylag_goose/Hungary/1941/2017	Central Agricultural Office Veterinary Diagnostic Directorate	Central Agricultural Office Veterinary Diagnostic Directorate
EPI954687	HA	Hungary	A/Mute swan/Hungary/1955/2017	Central Agricultural Office Veterinary Diagnostic Directorate	Central Agricultural Office Veterinary Diagnostic Directorate
EPI954695	HA	Hungary	A/Turkey/Hungary/2030/2017	Central Agricultural Office Veterinary Diagnostic Directorate	Central Agricultural Office Veterinary Diagnostic Directorate
EPI954703	HA	Hungary	A/Mute swan/Hungary/2193/2017	Central Agricultural Office Veterinary Diagnostic Directorate	Central Agricultural Office Veterinary Diagnostic Directorate
EPI954711	HA	Hungary	A/Mute swan/Hungary/2508/2017	Central Agricultural Office Veterinary Diagnostic Directorate	Central Agricultural Office Veterinary Diagnostic Directorate
EPI954719	HA	Hungary	A/Mute swan/Hungary/2825/2017	Central Agricultural Office Veterinary Diagnostic Directorate	Central Agricultural Office Veterinary Diagnostic Directorate
EPI954727	HA	Hungary	A/Mute swan/Hungary/3139/2017	Central Agricultural Office Veterinary Diagnostic Directorate	Central Agricultural Office Veterinary Diagnostic Directorate
EPI954735	HA	Hungary	A/Mute swan/Hungary/3513/2017	Central Agricultural Office Veterinary Diagnostic Directorate	Central Agricultural Office Veterinary Diagnostic Directorate
EPI954743	HA	Hungary	A/Mute swan/Hungary/3542/2017	Central Agricultural Office Veterinary Diagnostic Directorate	Central Agricultural Office Veterinary Diagnostic Directorate
EPI954751	HA	Hungary	A/Chicken/Hungary/2496/2017	National Food Chain Safety Office Veterinary Diagnostic Directorate Laboratory for Molecular Biology	Central Agricultural Office Veterinary Diagnostic Directorate
EPI954759	HA	Hungary	A/Peregrine_falcon/Hungary/4882/2017	Central Agricultural Office Veterinary Diagnostic Directorate	Central Agricultural Office Veterinary Diagnostic Directorate
EPI954767	HA	Hungary	A/Mute swan/Hungary/5316/2017	Central Agricultural Office Veterinary Diagnostic Directorate	Central Agricultural Office Veterinary Diagnostic Directorate
EPI954775	HA	Hungary	A/Common_buzzard/Hungary/7061/2017	Central Agricultural Office Veterinary Diagnostic Directorate	Central Agricultural Office Veterinary Diagnostic Directorate

Segment ID	Segment	Country	Isolate name	Originating Laboratory	Submitting Laboratory
EPI954783	HA	Hungary	A/Common_tern/Hungary/8187/2017	Central Agricultural Office Veterinary Diagnostic Directorate	Central Agricultural Office Veterinary Diagnostic Directorate
EPI954791	HA	Hungary	A/Harris_hawk/Hungary/2762a/2017	Central Agricultural Office Veterinary Diagnostic Directorate	Central Agricultural Office Veterinary Diagnostic Directorate
EPI954799	HA	Hungary	A/Harris_hawk/Hungary/2762b/2017	Central Agricultural Office Veterinary Diagnostic Directorate	Central Agricultural Office Veterinary Diagnostic Directorate
EPI954813	HA	Hungary	A/Greylag_goose/Hungary/320/2017	Central Agricultural Office Veterinary Diagnostic Directorate	Central Agricultural Office Veterinary Diagnostic Directorate
EPI954821	HA	Hungary	A/GuineaFowl/Hungary/596/2017	Central Agricultural Office Veterinary Diagnostic Directorate	Central Agricultural Office Veterinary Diagnostic Directorate
EPI954829	HA	Hungary	A/Chicken/Hungary/1751/2017	Central Agricultural Office Veterinary Diagnostic Directorate	Central Agricultural Office Veterinary Diagnostic Directorate
EPI954837	HA	Hungary	A/Mute swan/Hungary/3137/2017	Central Agricultural Office Veterinary Diagnostic Directorate	Central Agricultural Office Veterinary Diagnostic Directorate
EPI954845	HA	Hungary	A/Mallard/Hungary/5821/2017	Central Agricultural Office Veterinary Diagnostic Directorate	Central Agricultural Office Veterinary Diagnostic Directorate
EPI954861	HA	Hungary	A/Mallard/Hungary/1574b/2017	Central Agricultural Office Veterinary Diagnostic Directorate	Central Agricultural Office Veterinary Diagnostic Directorate
EPI954869	HA	Hungary	A/White_fronted_goose/Hungary/801/2017	Central Agricultural Office Veterinary Diagnostic Directorate	Central Agricultural Office Veterinary Diagnostic Directorate
EPI954877	HA	Hungary	A/Mallard/Hungary/1574a/2017	Central Agricultural Office Veterinary Diagnostic Directorate	Central Agricultural Office Veterinary Diagnostic Directorate
EPI956128	HA	Hungary	A/Goose/Hungary/59712/2016	Central Agricultural Office Veterinary Diagnostic Directorate	Central Agricultural Office Veterinary Diagnostic Directorate
EPI959523	HA	Hungary	A/Cormorant/Hungary/6102/2017	National Food Chain Safety Office Veterinary Diagnostic Directorate Laboratory for Molecular Biology	Central Agricultural Office Veterinary Diagnostic Directorate
EPI959531	HA	Hungary	A/Mute swan/Hungary/6092/2017	National Food Chain Safety Office Veterinary Diagnostic Directorate Laboratory for Molecular Biology	Central Agricultural Office Veterinary Diagnostic Directorate
EPI959539	HA	Hungary	A/Mute swan/Hungary/6276/2017	National Food Chain Safety Office Veterinary Diagnostic Directorate Laboratory for Molecular Biology	Central Agricultural Office Veterinary Diagnostic Directorate

Segment ID	Segment	Country	Isolate name	Originating Laboratory	Submitting Laboratory
EPI959547	HA	Hungary	A/Pheasant/Hungary/6553/2017	National Food Chain Safety Office Veterinary Diagnostic Directorate Laboratory for Molecular Biology	Central Agricultural Office Veterinary Diagnostic Directorate
EPI959555	HA	Hungary	A/Pheasant/Hungary/7685/2017	National Food Chain Safety Office Veterinary Diagnostic Directorate Laboratory for Molecular Biology	Central Agricultural Office Veterinary Diagnostic Directorate
EPI959563	HA	Hungary	A/Rook/Hungary/4975/2017	National Food Chain Safety Office Veterinary Diagnostic Directorate Laboratory for Molecular Biology	Central Agricultural Office Veterinary Diagnostic Directorate
EPI962046	HA	Hungary	A/Duck/Hungary/54738/2016	National Food Chain Safety Office Veterinary Diagnostic Directorate Laboratory for Molecular Biology	Central Agricultural Office Veterinary Diagnostic Directorate
EPI962056	HA	Hungary	A/Duck/Hungary/55764/2016	National Food Chain Safety Office Veterinary Diagnostic Directorate Laboratory for Molecular Biology	Central Agricultural Office Veterinary Diagnostic Directorate
EPI962066	HA	Hungary	A/Turkey/Hungary/53136/2016	National Food Chain Safety Office Veterinary Diagnostic Directorate Laboratory for Molecular Biology	Central Agricultural Office Veterinary Diagnostic Directorate
EPI858836	HA	India	A/duck/India/10CA01/2016	ICAR-National Institute of High Security Animal Diseases	ICAR-National Institute of High Security Animal Diseases
EPI858844	HA	India	A/painted stork/India/10CA03/2016	ICAR-National Institute of High Security Animal Diseases	ICAR-National Institute of High Security Animal Diseases
EPI517161	HA	Japan	A/Chicken/Kumamoto/1-7/2014	National Institute of Animal Health	National Agriculture and Food Research Organization
EPI548485	HA	Japan	A/duck/Chiba/26-372-48/2014	National Institute of Animal Health	National Institute of Animal Health
EPI548493	HA	Japan	A/duck/Chiba/26-372-61/2014	National Institute of Animal Health	National Institute of Animal Health
EPI553208	HA	Japan	A/crane/Kagoshima/KU1/2014	Kagoshima University	Kagoshima University
EPI553343	HA	Japan	A/chicken/Miyazaki/7/2014	NA	National Institute of Animal Health
EPI573638	HA	Japan	A/crane/Kagoshima/KU13/2014(H5N8)	Kagoshima University	Kagoshima University
EPI573646	HA	Japan	A/crane/Kagoshima/KU21/2014(H5N8)	Kagoshima University	Kagoshima University

Segment ID	Segment	Country	Isolate name	Originating Laboratory	Submitting Laboratory
EPI573654	HA	Japan	A/crane/Kagoshima/KU41/2014(H5N8)	Kagoshima University	Kagoshima University
EPI573664	HA	Japan	A/crane/Kagoshima/KU53/2015(H5N8)	Kagoshima University	Kagoshima University
EPI573672	HA	Japan	A/mallard duck/Kagoshima/KU70/2015(H5N8)	Kagoshima University	Kagoshima University
EPI573680	HA	Japan	A/mallard duck/Kagoshima/KU116/2015(H5N8)	Kagoshima University	Kagoshima University
EPI662624	HA	Japan	A/chicken/Saga/1-1/2015	National Institute of Animal Health	National Institute of Animal Health
EPI662632	HA	Japan	A/chicken/Okayama/1-2/2015	National Institute of Animal Health	National Institute of Animal Health
EPI662640	HA	Japan	A/chicken/Yamaguchi/6/2014	National Institute of Animal Health	National Institute of Animal Health
EPI662648	HA	Japan	A/chicken/Miyazaki/2-4/2014	National Institute of Animal Health	National Institute of Animal Health
EPI926621	HA	Kazakhstan	A/graylag goose/Kazakhstan/KR/2016	Research Institute of Experimental and Clinical Medicine	Research Institute of Experimental and Clinical Medicine
EPI573192	HA	Korea, Republic of	A/breeder chicken/Korea/H503/2014	Animal and Plant Quarantine Agency	Animal and Plant Quarantine Agency (QIA)
EPI573194	HA	Korea, Republic of	A/breeder chicken/Korea/H818/2014	NA	Animal and Plant Quarantine Agency (QIA)
EPI573195	HA	Korea, Republic of	A/breeder chicken/Korea/H818/2014	NA	Animal and Plant Quarantine Agency (QIA)
EPI573196	HA	Korea, Republic of	A/broiler duck/Korea/H651/2014	NA	Animal and Plant Quarantine Agency (QIA)
EPI573197	HA	Korea, Republic of	A/chicken/Korea/H881/2014	NA	Animal and Plant Quarantine Agency (QIA)
EPI573198	HA	Korea, Republic of	A/broiler duck/Korea/H959/2014	NA	Animal and Plant Quarantine Agency (QIA)

Segment ID	Segment	Country	Isolate name	Originating Laboratory	Submitting Laboratory
EPI573199	HA	Korea, Republic of	A/breeder chicken/Korea/H1068/2014	NA	Animal and Plant Quarantine Agency (QIA)
EPI573200	HA	Korea, Republic of	A/Korean native chicken/Korea/H1139/2014	NA	Animal and Plant Quarantine Agency (QIA)
EPI573201	HA	Korea, Republic of	A/chicken/Korea/H1236/2014	NA	Animal and Plant Quarantine Agency (QIA)
EPI573202	HA	Korea, Republic of	A/chicken/Korea/H1268/2014	Animal and Plant Quarantine Agency	Animal and Plant Quarantine Agency (QIA)
EPI573203	HA	Korea, Republic of	A/chicken/Korea/H1292/2014	NA	Animal and Plant Quarantine Agency (QIA)
EPI573204	HA	Korea, Republic of	A/goose/Korea/H1296/2014	NA	Animal and Plant Quarantine Agency (QIA)
EPI573205	HA	Korea, Republic of	A/Korean native chicken/Korea/H1299/2014	NA	Animal and Plant Quarantine Agency (QIA)
EPI573206	HA	Korea, Republic of	A/breeder duck/Korea/H1343/2014	NA	Animal and Plant Quarantine Agency (QIA)
EPI573207	HA	Korea, Republic of	A/chicken/Korea/H1350/2014	NA	Animal and Plant Quarantine Agency (QIA)
EPI573208	HA	Korea, Republic of	A/gadwall/Korea/H1351/2014	NA	Animal and Plant Quarantine Agency (QIA)
EPI573209	HA	Korea, Republic of	A/broiler duck/Korea/H1413/2014	NA	Animal and Plant Quarantine Agency (QIA)
EPI573210	HA	Korea, Republic of	A/broiler duck/Korea/H1414/2014	NA	Animal and Plant Quarantine Agency (QIA)
EPI573211	HA	Korea, Republic of	A/goose/Korea/H1545/2014	NA	Animal and Plant Quarantine Agency (QIA)
EPI573212	HA	Korea, Republic of	A/Korean native chicken/Korea/H1554/2014	NA	Animal and Plant Quarantine Agency (QIA)
EPI573213	HA	Korea, Republic of	A/broiler duck/Korea/H1556/2014	NA	Animal and Plant Quarantine Agency (QIA)

Segment ID	Segment	Country	Isolate name	Originating Laboratory	Submitting Laboratory
EPI573214	HA	Korea, Republic of	A/broiler duck/Korea/H1582/2014	NA	Animal and Plant Quarantine Agency (QIA)
EPI573215	HA	Korea, Republic of	A/breeder duck/Korea/H1596/2014	NA	Animal and Plant Quarantine Agency (QIA)
EPI573216	HA	Korea, Republic of	A/broiler duck/Korea/H1683/2014	NA	Animal and Plant Quarantine Agency (QIA)
EPI573217	HA	Korea, Republic of	A/broiler duck/Korea/H1685/2014	NA	Animal and Plant Quarantine Agency (QIA)
EPI573218	HA	Korea, Republic of	A/Korean native chicken/Korea/H1687/2014	NA	Animal and Plant Quarantine Agency (QIA)
EPI573219	HA	Korea, Republic of	A/goose/Korea/H1689/2014	NA	Animal and Plant Quarantine Agency (QIA)
EPI573220	HA	Korea, Republic of	A/goose/Korea/H1698/2014	NA	Animal and Plant Quarantine Agency (QIA)
EPI573221	HA	Korea, Republic of	A/broiler duck/Korea/H1731/2014	NA	Animal and Plant Quarantine Agency (QIA)
EPI573222	HA	Korea, Republic of	A/broiler duck/Korea/H1733/2014	NA	Animal and Plant Quarantine Agency (QIA)
EPI573223	HA	Korea, Republic of	A/broiler duck/Korea/H1734/2014	NA	Animal and Plant Quarantine Agency (QIA)
EPI573224	HA	Korea, Republic of	A/broiler duck/Korea/H1739/2014	NA	Animal and Plant Quarantine Agency (QIA)
EPI573225	HA	Korea, Republic of	A/broiler duck/Korea/H1745/2014	NA	Animal and Plant Quarantine Agency (QIA)
EPI573226	HA	Korea, Republic of	A/Korean native chicken/Korea/H1747/2014	NA	Animal and Plant Quarantine Agency (QIA)
EPI573227	HA	Korea, Republic of	A/breeder duck/Korea/H1752/2014	NA	Animal and Plant Quarantine Agency (QIA)
EPI573228	HA	Korea, Republic of	A/broiler duck/Korea/H1755/2014	NA	Animal and Plant Quarantine Agency (QIA)

Segment ID	Segment	Country	Isolate name	Originating Laboratory	Submitting Laboratory
EPI573229	HA	Korea, Republic of	A/broiler duck/Korea/H1763/2014	NA	Animal and Plant Quarantine Agency (QIA)
EPI573230	HA	Korea, Republic of	A/broiler duck/Korea/H1803/2014	NA	Animal and Plant Quarantine Agency (QIA)
EPI573231	HA	Korea, Republic of	A/broiler duck/Korea/H1839/2014	NA	Animal and Plant Quarantine Agency (QIA)
EPI573232	HA	Korea, Republic of	A/broiler duck/Korea/H1840/2014	NA	Animal and Plant Quarantine Agency (QIA)
EPI573233	HA	Korea, Republic of	A/Korean native chicken/Korea/H1847/2014	NA	Animal and Plant Quarantine Agency (QIA)
EPI573234	HA	Korea, Republic of	A/broiler duck/Korea/H1864/2014	NA	Animal and Plant Quarantine Agency (QIA)
EPI573235	HA	Korea, Republic of	A/Korean native chicken/Korea/H1903/2014	NA	Animal and Plant Quarantine Agency (QIA)
EPI573236	HA	Korea, Republic of	A/spot-billed duck/Korea/H1981/2014	NA	Animal and Plant Quarantine Agency (QIA)
EPI573237	HA	Korea, Republic of	A/mallard/Korea/H1991/2014	NA	Animal and Plant Quarantine Agency (QIA)
EPI573238	HA	Korea, Republic of	A/mallard/Korea/H2003/2014	NA	Animal and Plant Quarantine Agency (QIA)
EPI573239	HA	Korea, Republic of	A/mallard/Korea/H1924-6/2014	NA	Animal and Plant Quarantine Agency (QIA)
EPI573240	HA	Korea, Republic of	A/breeder duck/Korea/H345/2014	NA	Animal and Plant Quarantine Agency (QIA)
EPI573241	HA	Korea, Republic of	A/breeder duck/Korea/H566/2014	NA	Animal and Plant Quarantine Agency (QIA)
EPI573242	HA	Korea, Republic of	A/Common Teal/Korea/H844/2014	NA	Animal and Plant Quarantine Agency (QIA)
EPI595055	HA	Korea, Republic of	A/common teal/Korea/KU-12/2015	Avian diaseases laboratory, College of Veterinary Medicine, Konkuk University	Konkuk University

Segment ID	Segment	Country	Isolate name	Originating Laboratory	Submitting Laboratory
EPI595066	HA	Korea, Republic of	A/mallard/Korea/KU3-2/2015	Avian diaseases laboratory, College of Veterinary Medicine, Konkuk University	Konkuk University
EPI595079	HA	Korea, Republic of	A/mallard/Korea/N15-99/2015	Avian diaseases laboratory, College of Veterinary Medicine, Konkuk University	Konkuk University
EPI595082	HA	Korea, Republic of	A/mandarin duck/Korea/K14-363-1/2014	Avian diaseases laboratory, College of Veterinary Medicine, Konkuk University	Konkuk University
EPI595094	HA	Korea, Republic of	A/mandarin duck/Korea/K14-366-1/2014	Avian diaseases laboratory, College of Veterinary Medicine, Konkuk University	Konkuk University
EPI595107	HA	Korea, Republic of	A/mandarin duck/Korea/K14-367-1/2014	Avian diaseases laboratory, College of Veterinary Medicine, Konkuk University	Konkuk University
EPI595116	HA	Korea, Republic of	A/greater white-fronted goose/Korea/K14-367-4/2014	Avian diaseases laboratory, College of Veterinary Medicine, Konkuk University	Konkuk University
EPI595124	HA	Korea, Republic of	A/greater white-fronted goose/Korea/K14-369-3/2014	Avian diaseases laboratory, College of Veterinary Medicine, Konkuk University	Konkuk University
EPI595133	HA	Korea, Republic of	A/greater white-fronted goose/Korea/K14-371-4/2014	Avian diaseases laboratory, College of Veterinary Medicine, Konkuk University	Konkuk University
EPI595138	HA	Korea, Republic of	A/greater white-fronted goose/Korea/K14-372-2/2014	Avian diaseases laboratory, College of Veterinary Medicine, Konkuk University	Konkuk University
EPI595146	HA	Korea, Republic of	A/greater white-fronted goose/Korea/K14-374-1/2014	Avian diaseases laboratory, College of Veterinary Medicine, Konkuk University	Konkuk University
EPI837541	HA	Korea, Republic of	A/breeder duck/Korea/H2086/2015	NA	Animal and Plant Quarantine Agency (QIA)
EPI837542	HA	Korea, Republic of	A/mallard/Korea/H2102/2015	NA	Animal and Plant Quarantine Agency (QIA)
EPI837543	HA	Korea, Republic of	A/broiler duck/Korea/H2116/2015	NA	Animal and Plant Quarantine Agency (QIA)
EPI837544	HA	Korea, Republic of	A/broiler duck/Korea/H2194/2015	NA	Animal and Plant Quarantine Agency (QIA)
EPI837545	HA	Korea, Republic of	A/broiler duck/Korea/H2266/2015	NA	Animal and Plant Quarantine Agency (QIA)

Segment ID	Segment	Country	Isolate name	Originating Laboratory	Submitting Laboratory
EPI837546	HA	Korea, Republic of	A/broiler duck/Korea/H2278/2015	NA	Animal and Plant Quarantine Agency (QIA)
EPI837549	HA	Korea, Republic of	A/mallard/Korea/H2304/2015	NA	Animal and Plant Quarantine Agency (QIA)
EPI837550	HA	Korea, Republic of	A/mallard/Korea/H2321/2015	NA	Animal and Plant Quarantine Agency (QIA)
EPI837551	HA	Korea, Republic of	A/broiler duck/Korea/H2385/2015	NA	Animal and Plant Quarantine Agency (QIA)
EPI837552	HA	Korea, Republic of	A/broiler duck/Korea/H2393/2015	NA	Animal and Plant Quarantine Agency (QIA)
EPI837553	HA	Korea, Republic of	A/broiler duck/Korea/H2400/2015	NA	Animal and Plant Quarantine Agency (QIA)
EPI837554	HA	Korea, Republic of	A/broiler duck/Korea/H2405/2015	NA	Animal and Plant Quarantine Agency (QIA)
EPI837555	HA	Korea, Republic of	A/breeder chicken/Korea/H2496/2015	NA	Animal and Plant Quarantine Agency (QIA)
EPI837556	HA	Korea, Republic of	A/broiler duck/Korea/H2517/2015	NA	Animal and Plant Quarantine Agency (QIA)
EPI837557	HA	Korea, Republic of	A/broiler duck/Korea/H2531/2015	NA	Animal and Plant Quarantine Agency (QIA)
EPI837558	HA	Korea, Republic of	A/chicken/Korea/H2553/2015	NA	Animal and Plant Quarantine Agency (QIA)
EPI837559	HA	Korea, Republic of	A/chicken/Korea/H2565/2015	NA	Animal and Plant Quarantine Agency (QIA)
EPI837560	HA	Korea, Republic of	A/chicken/Korea/H2590/2015	NA	Animal and Plant Quarantine Agency (QIA)
EPI837561	HA	Korea, Republic of	A/korean native chicken/Korea/H2598/2015	NA	Animal and Plant Quarantine Agency (QIA)
EPI837562	HA	Korea, Republic of	A/broiler duck/Korea/H2606/2015	NA	Animal and Plant Quarantine Agency (QIA)

Segment ID	Segment	Country	Isolate name	Originating Laboratory	Submitting Laboratory
EPI837563	HA	Korea, Republic of	A/broiler duck/Korea/H2618/2015	NA	Animal and Plant Quarantine Agency (QIA)
EPI837564	HA	Korea, Republic of	A/duck/Korea/H2627/2015	NA	Animal and Plant Quarantine Agency (QIA)
EPI837565	HA	Korea, Republic of	A/duck/Korea/H2628/2015	NA	Animal and Plant Quarantine Agency (QIA)
EPI837566	HA	Korea, Republic of	A/broildr duck/Korea/H2637/2015	NA	Animal and Plant Quarantine Agency (QIA)
EPI837567	HA	Korea, Republic of	A/broiler duck/Korea/H2641/2015	NA	Animal and Plant Quarantine Agency (QIA)
EPI837568	HA	Korea, Republic of	A/broiler duck/Korea/H2649/2015	NA	Animal and Plant Quarantine Agency (QIA)
EPI837569	HA	Korea, Republic of	A/breeder duck/Korea/H2675/2015	NA	Animal and Plant Quarantine Agency (QIA)
EPI837570	HA	Korea, Republic of	A/broiler duck/Korea/H2826/2015	NA	Animal and Plant Quarantine Agency (QIA)
EPI837571	HA	Korea, Republic of	A/broiler duck/Korea/H2827/2015	NA	Animal and Plant Quarantine Agency (QIA)
EPI837572	HA	Korea, Republic of	A/broiler duck/Korea/LBM334/2015	NA	Animal and Plant Quarantine Agency (QIA)
EPI837573	HA	Korea, Republic of	A/mallard/Korea/H2891/2015	NA	Animal and Plant Quarantine Agency (QIA)
EPI837574	HA	Korea, Republic of	A/broiler duck/Korea/H2910/2015	NA	Animal and Plant Quarantine Agency (QIA)
EPI837575	HA	Korea, Republic of	A/broiler duck/Korea/H3078/2015	NA	Animal and Plant Quarantine Agency (QIA)
EPI837576	HA	Korea, Republic of	A/broiler duck/Korea/H3092/2015	NA	Animal and Plant Quarantine Agency (QIA)
EPI837577	HA	Korea, Republic of	A/broiler duck/Korea/H3093/2015	NA	Animal and Plant Quarantine Agency (QIA)

Segment ID	Segment	Country	Isolate name	Originating Laboratory	Submitting Laboratory
EPI837578	HA	Korea, Republic of	A/broiler duck/Korea/H3098/2015	NA	Animal and Plant Quarantine Agency (QIA)
EPI837579	HA	Korea, Republic of	A/broiler duck/Korea/H3104/2015	NA	Animal and Plant Quarantine Agency (QIA)
EPI837580	HA	Korea, Republic of	A/broiler duck/Korea/H3298/2015	NA	Animal and Plant Quarantine Agency (QIA)
EPI837581	HA	Korea, Republic of	A/broiler duck/Korea/H3301/2015	NA	Animal and Plant Quarantine Agency (QIA)
EPI837582	HA	Korea, Republic of	A/broiler duck/Korea/H3302/2015	NA	Animal and Plant Quarantine Agency (QIA)
EPI837583	HA	Korea, Republic of	A/breeder duck/Korea/16AQ17/2016	NA	Animal and Plant Quarantine Agency (QIA)
EPI837584	HA	Korea, Republic of	A/duck/Korea/16A02/2016	NA	Animal and Plant Quarantine Agency (QIA)
EPI952639	HA	Korea, Republic of	A/chicken/Korea/H903/2017	Avian disease division, Animal and Plant Quarantine Agency	Animal and Plant Quarantine Agency (QIA)
EPI952782	HA	Korea, Republic of	A/mallard duck/Korea/WA137/2017	Avian disease division, Animal and Plant Quarantine Agency	Animal and Plant Quarantine Agency (QIA)
EPI1019366	HA	Netherlands	A/Bk_swan/NL-Den Oever/16013973-002/2016	Wageningen Bioveterinary Reseach	Central Veterinary Institute
EPI1019374	HA	Netherlands	A/Bl_H_gull/NL-Slootdorp/16014102-002/2016	Wageningen Bioveterinary Reseach	Central Veterinary Institute
EPI1019382	HA	Netherlands	A/Buzzard/NL-Durgerdam/16015100-004/2016	Wageningen Bioveterinary Reseach	Central Veterinary Institute
EPI1019390	HA	Netherlands	A/C_Gull/NL-Slootdorp/16014102-003/2016	Wageningen Bioveterinary Reseach	Central Veterinary Institute
EPI1019398	HA	Netherlands	A/Ch/NL-Abbega/X16015736/2016	Wageningen Bioveterinary Reseach	Central Veterinary Institute
EPI1019406	HA	Netherlands	A/Ch/NL-Boven Leeuwen/16016151-006-010/2016	Wageningen Bioveterinary Reseach	Central Veterinary Institute

Segment ID	Segment	Country	Isolate name	Originating Laboratory	Submitting Laboratory
EPI1019414	HA	Netherlands	A/Ch/NL-Den Oever/16014231-001/2016	Wageningen Bioveterinary Reseach	Central Veterinary Institute
EPI1019422	HA	Netherlands	A/Ch/NL-Hiaure/16016112-001-005/2016	Wageningen Bioveterinary Reseach	Central Veterinary Institute
EPI1019430	HA	Netherlands	A/Ch/NL-Rhenen/16016141-006/2016	Wageningen Bioveterinary Reseach	Central Veterinary Institute
EPI1019438	HA	Netherlands	A/Ch/NL-Zoeterwoude/16016484-021-025/2016	Wageningen Bioveterinary Reseach	Central Veterinary Institute
EPI1019446	HA	Netherlands	A/Crow/NL-Oostwoud/16015372-004/2016	Wageningen Bioveterinary Reseach	Central Veterinary Institute
EPI1019454	HA	Netherlands	A/Dk/NL-Biddinghuizen/16014829-011-015/2016	Wageningen Bioveterinary Reseach	Central Veterinary Institute
EPI1019462	HA	Netherlands	A/Dk/NL-Biddinghuizen/16015083-016-020/2016	Wageningen Bioveterinary Reseach	Central Veterinary Institute
EPI1019470	HA	Netherlands	A/Dk/NL-Biddinghuizen/16015145-021-025/2016	Wageningen Bioveterinary Reseach	Central Veterinary Institute
EPI1019478	HA	Netherlands	A/Dk/NL-Kamperveen/16016104-001-005/2016	Wageningen Bioveterinary Reseach	Central Veterinary Institute
EPI1019486	HA	Netherlands	A/Dk/NL-Rotterdam/16014008-001-005/2016	Wageningen Bioveterinary Reseach	Central Veterinary Institute
EPI1019494	HA	Netherlands	A/Dk/NL-Stolwijk/16016291-016-020/2016	Wageningen Bioveterinary Reseach	Central Veterinary Institute
EPI1019502	HA	Netherlands	A/Eur_Wig/NL-Akkrum/16015817-003/2016	Wageningen Bioveterinary Reseach	Central Veterinary Institute
EPI1019510	HA	Netherlands	A/Eur_Wig/NL-De Waal (Texel)/16014891-003/2016	Wageningen Bioveterinary Reseach	Central Veterinary Institute
EPI1019518	HA	Netherlands	A/Eur_Wig/NL-De Waal (Texel)/16014891-004/2016	Wageningen Bioveterinary Reseach	Central Veterinary Institute
EPI1019526	HA	Netherlands	A/Eur_Wig/NL-Drieborg (Dollard)/16015513-001/2016	Wageningen Bioveterinary Reseach	Central Veterinary Institute

Segment ID	Segment	Country	Isolate name	Originating Laboratory	Submitting Laboratory
EPI1019534	HA	Netherlands	A/Eur_Wig/NL-Enumatil-Groningen/16015704-001/2016	Wageningen Bioveterinary Reseach	Central Veterinary Institute
EPI1019542	HA	Netherlands	A/Eur_Wig/NL-Ferwert/16015273-002/2016	Wageningen Bioveterinary Reseach	Central Veterinary Institute
EPI1019550	HA	Netherlands	A/Eur_Wig/NL-Gouda/16015824-001/2016	Wageningen Bioveterinary Reseach	Central Veterinary Institute
EPI1019558	HA	Netherlands	A/Eur_Wig/NL-Greonterp/16015653-001/2016	Wageningen Bioveterinary Reseach	Central Veterinary Institute
EPI1019566	HA	Netherlands	A/Eur_Wig/NL-Groningen/16015376-003/2016	Wageningen Bioveterinary Reseach	Central Veterinary Institute
EPI1019574	HA	Netherlands	A/Eur_Wig/NL-Leeuwarden/16015699-002/2016	Wageningen Bioveterinary Reseach	Central Veterinary Institute
EPI1019582	HA	Netherlands	A/Eur_Wig/NL-Leidschendam/16015697-007/2016	Wageningen Bioveterinary Reseach	Central Veterinary Institute
EPI1019590	HA	Netherlands	A/Eur_Wig/NL-Reeuwijk/16015903-003/2016	Wageningen Bioveterinary Reseach	Central Veterinary Institute
EPI1019598	HA	Netherlands	A/Eur_Wig/NL-Terschelling/16015692-010/2016	Wageningen Bioveterinary Reseach	Central Veterinary Institute
EPI1019606	HA	Netherlands	A/Eur_Wig/NL-Vianen/16015917-006/2016	Wageningen Bioveterinary Reseach	Central Veterinary Institute
EPI1019614	HA	Netherlands	A/Eur_Wig/NL-Walterswald/16015923-003/2016	Wageningen Bioveterinary Reseach	Central Veterinary Institute
EPI1019622	HA	Netherlands	A/Eur_Wig/NL-West Graftdijk/16015746-003/2016	Wageningen Bioveterinary Reseach	Central Veterinary Institute
EPI1019630	HA	Netherlands	A/Eur_Wig/NL-Wormer/16016143-002/2016	Wageningen Bioveterinary Reseach	Central Veterinary Institute
EPI1019638	HA	Netherlands	A/Eur_Wig/NL-Zoeterwoude/16015702-010/2016	Wageningen Bioveterinary Reseach	Central Veterinary Institute
EPI1019646	HA	Netherlands	A/Eur_Wig/NL-Zwolle/16015820-002/2016	Wageningen Bioveterinary Reseach	Central Veterinary Institute

Segment ID	Segment	Country	Isolate name	Originating Laboratory	Submitting Laboratory
EPI1019654	HA	Netherlands	A/G_c_grebe/NL-Monnickendam/16013865-009-010/2016	Wageningen Bioveterinary Reseach	Central Veterinary Institute
EPI1019662	HA	Netherlands	A/Go/NL-Roggebotsluis/16014462-010/2016	Wageningen Bioveterinary Reseach	Central Veterinary Institute
EPI1019670	HA	Netherlands	A/Gr_bk_bd_gull/NL-Slootdorp/16014102-005/2016	Wageningen Bioveterinary Reseach	Central Veterinary Institute
EPI1019678	HA	Netherlands	A/Grey_Go/NL-Groot-Ammers/16015901-012/2016	Wageningen Bioveterinary Reseach	Central Veterinary Institute
EPI1019686	HA	Netherlands	A/Gull/NL-Marker Wadden/16014466-020/2016	Wageningen Bioveterinary Reseach	Central Veterinary Institute
EPI1019694	HA	Netherlands	A/Gull1/NL-Marker Wadden/16014466-011/2016	Wageningen Bioveterinary Reseach	Central Veterinary Institute
EPI1019702	HA	Netherlands	A/Gull10/NL-Marker Wadden/16014466-014/2016	Wageningen Bioveterinary Reseach	Central Veterinary Institute
EPI1019710	HA	Netherlands	A/L-bl-ba-gull/NL-Sovon/16014324-014/2016	Wageningen Bioveterinary Reseach	Central Veterinary Institute
EPI1019718	HA	Netherlands	A/M_Swan/NL-Roggebotsluis/16014462-019/2016	Wageningen Bioveterinary Reseach	Central Veterinary Institute
EPI1019726	HA	Netherlands	A/Magpie/NL-Volendam/16014331-002/2016	Wageningen Bioveterinary Reseach	Central Veterinary Institute
EPI1019734	HA	Netherlands	A/Mal/NL-IJsselmuiden/16015448-002/2016	Wageningen Bioveterinary Reseach	Central Veterinary Institute
EPI1019742	HA	Netherlands	A/Mal/NL-Mastenbroek/16015378-002/2016	Wageningen Bioveterinary Reseach	Central Veterinary Institute
EPI1019750	HA	Netherlands	A/P_falcon/NL-Vrouwenpolder (Zeeland)/16015510-001/2016	Wageningen Bioveterinary Reseach	Central Veterinary Institute
EPI1019758	HA	Netherlands	A/Sea_eagle/NL-Assen/16015398-002/2016	Wageningen Bioveterinary Reseach	Central Veterinary Institute
EPI1019766	HA	Netherlands	A/T_Dk/NL-Almeerder Zand/16014341-003/2016	Wageningen Bioveterinary Reseach	Central Veterinary Institute

Segment ID	Segment	Country	Isolate name	Originating Laboratory	Submitting Laboratory
EPI1019774	HA	Netherlands	A/T_Dk/NL-Monnickendam/16013865-006-008/2016	Wageningen Bioveterinary Reseach	Central Veterinary Institute
EPI1019782	HA	Netherlands	A/T_Dk/NL-Roggebotsluis/16014462-015/2016	Wageningen Bioveterinary Reseach	Central Veterinary Institute
EPI1019790	HA	Netherlands	A/T_Dk/NL-Rotterdam/16014155-001/2016	Wageningen Bioveterinary Reseach	Central Veterinary Institute
EPI1019798	HA	Netherlands	A/T_Dk/NL-Werkendam/16014159-002/2016	Wageningen Bioveterinary Reseach	Central Veterinary Institute
EPI1019806	HA	Netherlands	A/T_Dk/NL-Werkendam/16014159-003/2016	Wageningen Bioveterinary Reseach	Central Veterinary Institute
EPI1019814	HA	Netherlands	A/T_Dk/NL-Zeewolde/16013976-001/2016	Wageningen Bioveterinary Reseach	Central Veterinary Institute
EPI1019822	HA	Netherlands	A/T_Dk/NL-Zeewolde/16013976-001-003/2016	Wageningen Bioveterinary Reseach	Central Veterinary Institute
EPI1019830	HA	Netherlands	A/T_Dk/NL-Zeewolde/16013976-004/2016	Wageningen Bioveterinary Reseach	Central Veterinary Institute
EPI1019838	HA	Netherlands	A/T_Dk/NL-Zeewolde/16013976-004-006/2016	Wageningen Bioveterinary Reseach	Central Veterinary Institute
EPI1019846	HA	Netherlands	A/T_Dk/NL-Zeewolde/16013976-005/2016	Wageningen Bioveterinary Reseach	Central Veterinary Institute
EPI1019854	HA	Netherlands	A/T_Dk/NL-Zeewolde/16013976-006/2016	Wageningen Bioveterinary Reseach	Central Veterinary Institute
EPI1019862	HA	Netherlands	A/T_Dk/NL-Zuidoost Beemster/16014148-002/2016	Wageningen Bioveterinary Reseach	Central Veterinary Institute
EPI1019870	HA	Netherlands	A/T_Dk/NL-Zuidoost Beemster/16014148-009/2016	Wageningen Bioveterinary Reseach	Central Veterinary Institute
EPI1019878	HA	Netherlands	A/Teal/NL-Ferwert/16015273-013/2016	Wageningen Bioveterinary Reseach	Central Veterinary Institute
EPI547678	HA	Netherlands	A/Chicken/Netherlands/14015526/2014	Wageningen Bioveterinary Research	Wageningen Bioveterinary Research

Segment ID	Segment	Country	Isolate name	Originating Laboratory	Submitting Laboratory
EPI548623	HA	Netherlands	A/chicken/Netherlands/14015531/2014	Wageningen Bioveterinary Research	Wageningen Bioveterinary Research
EPI551143	HA	Netherlands	A/eurasian wigeon/Netherlands/emc-1/2014	Erasmus Medical Center	Erasmus Medical Center
EPI551149	HA	Netherlands	A/eurasian wigeon/Netherlands/emc-2/2014	Erasmus Medical Center	Erasmus Medical Center
EPI552760	HA	Netherlands	A/eurasian wigeon/Netherlands/emc-1/2014	Erasmus Medical Center	Erasmus Medical Center
EPI552768	HA	Netherlands	A/eurasian wigeon/Netherlands/emc-2/2014	Erasmus Medical Center	Erasmus Medical Center
EPI552776	HA	Netherlands	A/chicken/Netherlands/emc-3/2014	Erasmus Medical Center	Erasmus Medical Center
EPI573163	HA	Netherlands	A/chicken/Netherlands/14015766/2014	Wageningen Bioveterinary Research	Wageningen Bioveterinary Research
EPI573171	HA	Netherlands	A/Chicken/Netherlands/14015824/2014	Wageningen Bioveterinary Research	Wageningen Bioveterinary Research
EPI573179	HA	Netherlands	A/duck/Netherlands/14015898/2014	Wageningen Bioveterinary Research	Wageningen Bioveterinary Research
EPI573187	HA	Netherlands	A/chicken/Netherlands/14016437/2014	Wageningen Bioveterinary Research	Wageningen Bioveterinary Research
EPI585111	HA	Netherlands	A/eurasian wigeon/Netherlands/1/2015	Erasmus Medical Center	Erasmus Medical Center
EPI873659	HA	Netherlands	A/Eurasian wigeon/Netherlands/1/2016	Erasmus Medical Center	Erasmus Medical Center
EPI959407	HA	Netherlands	A/Eurasian Wigeon/Netherlands/2/2016	Erasmus Medical Center	Erasmus Medical Center
EPI860231	HA	Poland	A/wild duck/Poland/82A/2016	NA	National Veterinary Research Institut Poland, PIWet-PIB
EPI869924	HA	Poland	A/domestic goose/Poland/33/2016	NA	National Veterinary Research Institut Poland, PIWet-PIB

Segment ID	Segment	Country	Isolate name	Originating Laboratory	Submitting Laboratory
EPI869926	HA	Poland	A/domestic goose/Poland/72/2016	NA	National Veterinary Research Institut Poland, PIWet-PIB
EPI869927	HA	Poland	A/chicken/Poland/79A/2016	NA	National Veterinary Research Institut Poland, PIWet-PIB
EPI869928	HA	Poland	A/chicken/Poland/85A/2016	NA	National Veterinary Research Institut Poland, PIWet-PIB
EPI869929	HA	Poland	A/mute swan/Poland/108/2016	NA	National Veterinary Research Institut Poland, PIWet-PIB
EPI869930	HA	Poland	A/turkey/Poland/78/2016	NA	National Veterinary Research Institut Poland, PIWet-PIB
EPI869931	HA	Poland	A/turkey/Poland/83/2016	NA	National Veterinary Research Institut Poland, PIWet-PIB
EPI869940	HA	Poland	A/herring gull/Poland/84/2016	NA	National Veterinary Research Institut Poland, PIWet-PIB
EPI959433	HA	Poland	A/chicken/Poland/114/2016	NA	National Veterinary Research Institut Poland, PIWet-PIB
EPI959435	HA	Poland	A/chicken/Poland/002/2017	NA	National Veterinary Research Institut Poland, PIWet-PIB
EPI1044550	HA	Russian Federation	A/mute swan/Kaliningrad/132/2017	NA	State Research Center of Virology and Biotechnology (VECTOR)
EPI1045597	HA	Russian Federation	A/chicken/Shchyolkovo/47/2017	NA	State Research Center of Virology and Biotechnology (VECTOR)
EPI1045611	HA	Russian Federation	A/chicken/Rostov/44/2017	NA	State Research Center of Virology and Biotechnology (VECTOR)
EPI1045619	HA	Russian Federation	A/chicken/Tatarstan/88/2017	NA	State Research Center of Virology and Biotechnology (VECTOR)
EPI553349	HA	Russian Federation	A/wigeon/Sakha/1/2014	State Research Center of Virology and Biotechnology (VECTOR)	State Research Center of Virology and Biotechnology (VECTOR)
EPI773757	HA	Russian Federation	A/great crested grebe/Uvs-Nuur Lake/341/2016	Research Institute of Experimental and Clinical Medicine	Research Institute of Experimental and Clinical Medicine

Segment ID	Segment	Country	Isolate name	Originating Laboratory	Submitting Laboratory
EPI823460	HA	Russian Federation	A/great crested grebe/Tyva/34/2016	State Research Center of Virology and Biotechnology (VECTOR)	WHO National Influenza Centre Russian Federation
EPI823748	HA	Russian Federation	A/wild duck/Tyva/35/2016	State Research Center of Virology and Biotechnology (VECTOR)	WHO National Influenza Centre Russian Federation
EPI823756	HA	Russian Federation	A/black-headed gull/Tyva/41/2016	State Research Center of Virology and Biotechnology (VECTOR)	WHO National Influenza Centre Russian Federation
EPI836606	HA	Russian Federation	A/grey heron /Uvs-Nuur Lake/20/2016	Research Institute of Experimental and Clinical Medicine	Research Institute of Experimental and Clinical Medicine
EPI836614	HA	Russian Federation	A/common tern /Uvs-Nuur Lake/26/2016	Research Institute of Experimental and Clinical Medicine	Research Institute of Experimental and Clinical Medicine
EPI869936	HA	Russian Federation	A/chicken/Kalmykia/2661/2016	NA	State Research Center of Virology and Biotechnology (VECTOR)
EPI869938	HA	Russian Federation	A/chicken/Astrakhan/3131/2016	NA	State Research Center of Virology and Biotechnology (VECTOR)
EPI909380	HA	Russian Federation	A/long-eared owl/Voronezh/15/2017	NA	State Research Center of Virology and Biotechnology (VECTOR)
EPI909388	HA	Russian Federation	A/Ural owl/Voronezh/14/2017	NA	State Research Center of Virology and Biotechnology (VECTOR)
EPI909396	HA	Russian Federation	A/long-eared owl/Voronezh/16/2017	NA	State Research Center of Virology and Biotechnology (VECTOR)
EPI909404	HA	Russian Federation	A/chicken/Voronezh/18/2017	NA	State Research Center of Virology and Biotechnology (VECTOR)
EPI909412	HA	Russian Federation	A/chicken/Voronezh/19/2017	NA	State Research Center of Virology and Biotechnology (VECTOR)
EPI909420	HA	Russian Federation	A/chicken/Voronezh/20/2017	NA	State Research Center of Virology and Biotechnology (VECTOR)
EPI909428	HA	Russian Federation	A/turkey/Rostov/11/2017	NA	State Research Center of Virology and Biotechnology (VECTOR)
EPI909436	HA	Russian Federation	A/goose/Krasnodar/3144/2017	NA	State Research Center of Virology and Biotechnology (VECTOR)

Segment ID	Segment	Country	Isolate name	Originating Laboratory	Submitting Laboratory
EPI909444	HA	Russian Federation	A/mute swan/Krasnodar/25/2017	NA	State Research Center of Virology and Biotechnology (VECTOR)
EPI909452	HA	Russian Federation	A/wild duck/Tatarstan/3059/2016	NA	State Research Center of Virology and Biotechnology (VECTOR)
EPI909460	HA	Russian Federation	A/chicken/Kalmykia/2643/2016	NA	State Research Center of Virology and Biotechnology (VECTOR)
EPI925956	HA	Russian Federation	A/gadwall/Chany/97/2016	Research Institute of Experimental and Clinical Medicine	WHO National Influenza Centre Russian Federation
EPI926605	HA	Russian Federation	A/domestic duck/Siberia/50K/2016	NA	Research Institute of Experimental and Clinical Medicine
EPI926613	HA	Russian Federation	A/domestic duck/Siberia/103/2016	Research Institute of Experimental and Clinical Medicine	Research Institute of Experimental and Clinical Medicine
EPI961449	HA	Russian Federation	A/gadwall/Kurgan/2442/2016	NA	State Research Center of Virology and Biotechnology (VECTOR)
EPI961459	HA	Russian Federation	A/chicken/Sergiyev Posad/38/2017	NA	State Research Center of Virology and Biotechnology (VECTOR)
EPI961467	HA	Russian Federation	A/chicken/Sergiyev Posad/39/2017	NA	State Research Center of Virology and Biotechnology (VECTOR)
EPI576384	HA	Sweden	A/MuteSwan/Sweden/SVA150311KU0277/SZ502/2015	National Veterinary Institute	National Veterinary Institute
EPI576391	HA	Sweden	A/MuteSwan/Sweden/SVA150313KU0141/SZ543/2015	National Veterinary Institute	National Veterinary Institute
EPI863826	HA	Sweden	A/Chicken/Sweden/SVA161122KU0453/SZ0209316/2016	National Veterinary Institute	National Veterinary Institute
EPI863834	HA	Sweden	A/Chicken/Sweden/SVA161122KU0453/SZ0209317/2016	National Veterinary Institute	National Veterinary Institute
EPI863849	HA	Sweden	A/Chicken/Sweden/SVA161122KU0453/SZ0209318/2016	National Veterinary Institute	National Veterinary Institute
EPI863857	HA	Sweden	A/Chicken/Sweden/SVA161122KU0453/SZ0209321/2016	National Veterinary Institute	National Veterinary Institute

Segment ID	Segment	Country	Isolate name	Originating Laboratory	Submitting Laboratory
EPI863865	HA	Sweden	A/Common Goldeneye/Sweden/SVA161117KU0322/SZ0002165/2016	National Veterinary Institute	National Veterinary Institute
EPI969254	HA	Switzerland	A/Tufted Duck/Switzerland/V237/2016	Institute of Virology and Immunology IVI	Faculty of Veterinary Medicine at the University of Bern
EPI588952	HA	Taiwan	A/goose/Taiwan/a015/2015	Animal Health Research Institute	Animal Health Research Institute
EPI588976	HA	Taiwan	A/duck/Taiwan/a068/2015	Animal Health Research Institute	Animal Health Research Institute
EPI961933	HA	Taiwan	A/chicken/Taiwan/x37/2016	NA	Animal Health Research Institute
EPI547673	HA	United Kingdom	A/duck/England/36254/14	Animal and Plant Health Agency (APHA)	Animal and Plant Health Agency (APHA)
EPI550848	HA	United Kingdom	A/duck/England/36038/14	Animal and Plant Health Agency (APHA)	Animal and Plant Health Agency (APHA)
EPI550849	HA	United Kingdom	A/duck/England/36226/14	Animal and Plant Health Agency (APHA)	Animal and Plant Health Agency (APHA)
EPI868848	HA	United Kingdom	A/turkey/England/052131/2016	Animal and Plant Health Agency (APHA)	Animal and Plant Health Agency (APHA)
EPI942935	HA	United Kingdom	A/turkey/England/003778/2017	Animal and Plant Health Agency (APHA)	Animal and Plant Health Agency (APHA)
EPI942943	HA	United Kingdom	A/chicken/Wales/000023/2016	Animal and Plant Health Agency (APHA)	Animal and Plant Health Agency (APHA)
EPI943320	HA	United Kingdom	A/pochard_duck/England/SA12_157809/2016	Animal and Plant Health Agency (APHA)	Animal and Plant Health Agency (APHA)
